# VviNAC33 promotes organ de‐greening and represses vegetative growth during the vegetative‐to‐mature phase transition in grapevine

**DOI:** 10.1111/nph.17263

**Published:** 2021-03-16

**Authors:** Erica D’Incà, Stefano Cazzaniga, Chiara Foresti, Nicola Vitulo, Edoardo Bertini, Mary Galli, Andrea Gallavotti, Mario Pezzotti, Giovanni Battista Tornielli, Sara Zenoni

**Affiliations:** ^1^ Department of Biotechnology University of Verona Verona 37134 Italy; ^2^ Waksman Institute of Microbiology Rutgers University Piscataway NJ 08854‐8020 USA

**Keywords:** DAP‐seq, de‐greening, grapevine, NAC33, phase transition, transcriptomics, vegetative growth

## Abstract

- Plants undergo several developmental transitions during their life cycle. In grapevine, a perennial woody fruit crop, the transition from vegetative/green‐to‐mature/woody growth involves transcriptomic reprogramming orchestrated by a small group of genes encoding regulators, but the underlying molecular mechanisms are not fully understood.
- We investigated the function of the transcriptional regulator *VviNAC33* by generating and characterizing transgenic overexpressing grapevine lines and a chimeric repressor, and by exploring its putative targets through a DNA affinity purification sequencing (DAP‐seq) approach combined with transcriptomic data.
- We demonstrated that VviNAC33 induces leaf de‐greening, inhibits organ growth and directly activates the expression of *STAY‐GREEN PROTEIN 1* (*SGR1*), which is involved in Chl and photosystem degradation, and *AUTOPHAGY 8f* (*ATG8f*), which is involved in the maturation of autophagosomes. Furthermore, we show that VviNAC33 directly inhibits *AUXIN EFFLUX FACILITATOR PIN1*, *RopGEF1* and *ATP SYNTHASE GAMMA CHAIN 1T* (*ATPC1*), which are involved in photosystem II integrity and activity.
- Our results show that VviNAC33 plays a major role in terminating photosynthetic activity and organ growth as part of a regulatory network governing the vegetative‐to‐mature phase transition.

## Abstract 2

See also the Commentary on this article by Kelly & Allan, **231**: 505–507.

## Introduction

Plants develop through a succession of distinct growth phases, and the correct timing of the transitions is important for plant fitness and agronomic performance (Demura & Ye, 2010). In seed plants, shoots pass through a vegetative growth phase, further divided into juvenile and adult stages, characterized by an increase in photosynthetic capacity and organ size. A reproductive phase follows, during which the vegetative shoot apical meristem assumes an inflorescence meristem identity (Huijser & Schmid, 2011). An essential transition for survival under adverse environmental conditions is the shift from an active to a dormant state, involving the cessation of growth, the repression of auxin responses, cell wall thickening, and adaptations to survive cold temperatures. For example, seed dormancy prevents germination out of season, whereas bud dormancy and shoot lignification in perennial woody plants allow overwinter survival (Schrader *et* *al*., 2004).

In fleshy fruit species, the transition from unripe fruit (which must be protected against herbivory) to ripe fruit (which must appeal to the same herbivores to facilitate seed dispersal) is another dramatic shift in survival strategy. Fruit ripening involves a tightly coordinated sequence of chemical and physiological alterations (Giovannoni *et* *al*., 2017).

Senescence is the last part of the plant developmental program before cell death (Woo *et* *al*., 2013). The transition to senescence involves the ordered disassembly of molecules and cellular components that accumulated during vegetative growth (Lim *et* *al*., 2007). The earliest and most significant change in cell structure is the disassembly of the chloroplast, an organelle that contains up to 70% of the total leaf protein content. Like fruit ripening, leaf senescence is a complex genetic and epigenetic program that integrates endogenous developmental signals and environmental cues.

In recent years it has become evident that the genetic networks underlying these phase transitions share certain common factors. For example, the juvenile‐to‐adult and vegetative‐to‐reproductive phase transitions involve two evolutionarily conserved microRNAs (*miR156* and *miR172*) and their targets (Huijser & Schmid, 2011; Ma *et* *al*., 2020). Recently, certain NAM/ATAF/CUC (NAC) transcription factors were found to be key regulators of both fruit ripening and senescence, such as tomato NOR, a well‐characterized master regulator of the onset of fruit ripening and a positive regulator of leaf senescence (Ma *et* *al*., 2019).

NAC proteins form a large plant‐specific family of transcription factors (TFs) that control the expression of target genes by forming heterodimers with other NAC proteins as well as other TF families (Kim *et* *al*., 2016). The analysis of *NAC* genes in different crops has shed light on their role in integrating developmental age and environmental signals during plant development (Liang *et* *al*., 2014; Podzimska‐Sroka *et* *al*., 2015). For example, genetic studies have identified ANAC092/ORESARA1 (ORE1), ANAC029/Arabidopsis NAC‐LIKE Activated by AP3/PI (AtNAP), ANAC059/ORESARA1 SISTER1 (ORS1), and ANAC016 as positive regulators of leaf senescence (Guo & Gan, 2006; Kim *et* *al*., 2009; Balazadeha *et* *al*., 2011; Sakuraba *et* *al*., 2016), whereas ANAC042/JUNGBRUNNEN1 (JUB1) and ANAC083/VND‐INTERACTING2 (VNI2) are negative regulators (Yang *et* *al*., 2011; Wu *et* *al*., 2012). ANAC092/ORE1 is considered to be the key positive regulator of leaf senescence, involved in a delicately balanced feed‐forward loop that promotes ethylene‐mediated Chl degradation.

Grapevine (*Vitis* *vinifera*) is an important perennial fruit crop and a useful model of developmental phase transition in woody species, featuring all the major developmental and physiological processes and benefiting from a well characterized fruit development process. We previously revealed major transcriptomic reprogramming in all of the organs and tissues during the shift from vegetative/green‐to‐mature/woody development (Fasoli *et* *al*., 2012). Co‐expression network analysis uncovered a small group of genes that potentially act as switches for this phase transition (Palumbo *et* *al*., 2014; Massonnet *et* *al*., 2017), including the NAC family member *VviNAC33*, whose sudden expression was among the earliest molecular signals of the onset of berry ripening (Fasoli *et* *al*., 2018). Preliminary results indicate that *VviNAC33* is controlled by two transcription factors, VvibHLH075 and VviWRKY19, which have been identified previously as potential regulators of berry phase transition (Fasoli *et* *al*., 2018).

Here we characterize the activity of VviNAC33 by characterizing transient and stable overexpression lines and the creation of a chimeric repressor *in* *planta*. We also used DNA affinity purification sequencing (DAP‐seq) coupled with transcriptomic analysis to identify putative direct targets of VviNAC33, revealing that it acts as an activator and repressor of transcription. We propose that VviNAC33 functions in a regulative network controlling the de‐greening and the suppression of organ growth during the transition from the vegetative to the mature phase of development.

## Materials and Methods

### Plant material

*Nicotiana* *benthamiana* Domin plants, *Vitis* *vinifera* L. cv Sultana plantlets, cv Syrah embryogenic calli for the *VviNAC33* genetic transformation, cv Garganega embryogenic calli for the *VviNAC33‐EAR* genetic transformation, *VviNAC33* and *VviNAC33‐EAR* transgenic plants were grown as described in Amato *et* *al*. (2019). For the DAP‐seq analysis, cv Syrah fruiting cuttings were propagated as described by Mullins & Rajasekaran (1981).

### Protoplast isolation

Protoplasts were prepared from 0.5 g of leaf tissue as described by Zhao *et* *al*. (2016). For VviNAC33 subcellular localization, protoplasts were isolated from a pool of four leaves each from three cv Corvina plants. For chloroplast distribution analysis, protoplasts were isolated from a pool created by four fully expanded leaves, each taken from three plants of every transgenic overexpressing line plus a control.

### Bioinformatics

NAC protein sequences from different species were aligned using muscle with default settings. The unrooted phylogenetic tree was constructed in Mega v.7.0 (http://www.megasoftware.net/; Kumar *et* *al*., 2016) using the neighbour‐joining method based on the following parameters: pairwise deletion and bootstrap analysis with 1000 replicates.

Conserved motifs were identified using the meme suite (http://memesuite.org/tools/meme; Bailey *et* *al*., 2009) with standard parameters, a maximum of 20 motifs and an optimum motif width of 4–60 amino acids. Transcriptomic data for gene expression analysis were retrieved from the Corvina atlas and two berry‐specific expression maps (Fasoli *et* *al*., 2012, 2018; Massonnet *et* *al*., 2017). Co‐expression analysis based on Pearson’s correlation coefficient was carried out using Corto software and *VviNAC33* (VIT_19s0027g00230) as the bait in the Corvina atlas. shinygo v.0.61 was used for enrichment analysis (Ge *et* *al*., 2020).

### Isolation and cloning

The *VviNAC33* coding sequence (with 3′ UTR) and *VviNAC33‐EAR* were amplified from cv Corvina mid‐ripening and ripening berry skin using KAPA HiFi DNA polymerase (KAPA Biosystems, Wilmington, MA, USA); the primers used are listed in Supporting Information Table S1. The polymerase chain reaction (PCR) products were directionally cloned into the Gateway entry vector pENTR/D‐TOPO (Invitrogen, Thermo Fisher Scientific, Waltham, MA, USA).

For agroinfiltration in *N*. *benthamiana* and the transient expression, the *VviNAC33* sequence was transferred into the binary overexpression vector pK7GW2.0 (Laboratory of Plant Systems Biology, Ghent University, Belgium) by site‐specific LR recombination. For stable overexpression, the sequence was transferred to the modified binary vector pK7GW2.0 containing an eGFP expression cassette driven by the Arabidopsis ubiquitin 10 promoter. The *VviNAC33‐EAR* chimeric repressor was transferred into the modified pK7WG2 vector, harboring the 1320‐bp endogenous *VviNAC33* promoter (isolated from cv Corvina genomic DNA) instead of the *cauliflower mosaic virus (CaMV)* *35S* promoter.

For subcellular localization, the green fluorescent protein (GFP) sequence was fused to the C‐terminus of *VviNAC33*.

### Subcellular localization

Protoplasts were transfected with 50 µg of the *pK7FWG2: VviNAC33* vector or the control vector (*pK7FWG2: eGFP*) as described by Amato *et* *al*. (2019).

### DAP‐seq

Genomic DNA was extracted from 1 g of ground young cv Syrah leaves as described by Thomas & Scott (1993), and the Illumina libraries were prepared as described in Galli *et* *al*. (2018). The *VviNAC33* sequence was transferred from the pENTR/D‐TOPO to the Gateway‐compatible destination vector pIX‐HALO (Bartlett *et* *al*., 2017). The HALO‐VviNAC33 and GST‐HALO (used as negative control) fusion proteins were *in* *vitro* translated using the TNT^R^ SP6 coupled reticulocyte lysate system (Promega). The DAP‐seq was performed as described by Galli *et* *al*. (2018) and a total of 3.5 million of 75 bp single reads were obtained for each sample.

### Read mapping, filtering, peak calling and motif analysis

fastq files were trimmed using trimmomatic (Bolger *et* *al*., 2014) with the following parameters: ILLUMINACLIP = TruSeq3–, SE = 2:30:10. LEADING = 3, TRAILING = 3, SLIDINGWINDOW = 4:20, MINLEN = 50. Trimmed reads were mapped to the grape reference genome v.12X.2 (nuclear chromosomes only) at Urgi (https://urgi.versailles.inra.fr/Species/Vitis/Annotations) using bowtie2 v.2.3.4.3 (Langmead & Salzberg, 2012). Reads mapping to multiple locations were filtered, removing all reads with the XS:i field present in the BAM file. Uniquely mapped reads were used for all subsequent steps. Peaks were called using Gem v.3.4 (Guo *et* *al*., 2012) and the GST‐HALO negative control sample for background subtraction, applying the following parameters: ‐‐d Read_Distribution_default.txt ‐‐k_min 6 ‐‐k_max 20 ‐‐outNP. To reduce the number of false positives, we removed all peaks with a sample/control ratio < 5‐fold. The remaining peaks were associated with nearest genes using the chipseeker R package (Yu *et* *al*., 2015). For gene annotation, we used the V1 on the 12X.0 assembly transposed to the 12X.2 assembly. The gff3 files were downloaded from Unité de Recherche Génomique Info (URGI). For visualization in the Integrative Genome Browser (IGV), bam files were converted to bigwig files using deeptools v.3.4.3, bamCoverage with a 10‐bp bin size, and fragments per kilobase per million reads (FPKM) normalization. Motifs were detected and assigned using RSAT Plants NGS ChIP‐Seq peak‐motifs analysis (http://rsat.eead.csic.es/plants/peak‐motifs_form.cgi) and RSAT Plants Motif Discovery dyad‐analysis (http://rsat.eead.csic.es/plants/dyad‐analysis_form.cgi).

### Transient expression

For *N*. *benthamiana*, pK7WG2.0 vectors containing *35S: VviNAC33* or a noncoding sequence (negative control) were transferred to *Agrobacterium* *tumefaciens* strain C58C1 by electroporation (Hellens *et* *al*., 2000) and three fully expanded leaves each from four plants were syringe infiltrated for both vectors. Phenotypic analysis was carried out 3 d after agroinfiltration.

The same vectors were used for transient expression in cv Sultana. Five‐week‐old *in* *vitro* plantlets (eight plants for *VviNAC33* overexpression and seven for the control) were vacuum infiltrated as previously described (Amato *et* *al*., 2017). Molecular analyses were carried out on leaf samples collected 7 d after the agroinfiltration.

### Transgenic plants

The pK7WG2.0 vectors containing *35S: VviNAC33*, *VviNAC33‐EAR* or the *eGFP* sequence (negative control) were transferred to *A*. *tumefaciens* strain EHA105 by electroporation. The genetic transformations were performed as described by Amato *et* *al*. (2019).

### Leaf area measurement

Leaf area was estimated as proposed by Smith & Kliewer (1984) by measuring maximum length and width of three laminae of mature leaves (at the 6^th^/7^th^ node from apex) each from four plants per transgenic line plus control. Mean ± SD of 12 biological replicates was calculated.

### Pigment analysis and photosynthetic parameters

For *N*. *Benthamiana* four biological replicates, each consisting of a pool of infiltrated leaves from each plant, were analyzed. For the OXNAC33 and EARNAC33 transgenic plants, four biological replicates each consisting of a pool of three young leaves from each of the OX1, 2, and 3 lines plus control, and four biological replicates each consisting of a pool of three fully expanded leaves for each of the EAR1, 2, and 3 lines plus control were analyzed. Pigments were measured according to Cazzaniga *et* *al*. (2020).

Photosynthetic parameters and OJIP curves were measured using a DUAL‐PAM‐100 fluorimeter (Heinz–Walz, Effeltrich, Germany) as previously described (Baker, 2008). Proton motive force was measured by electrochromic shift (ECS) using a Multispeq v.2.0 (PhotosynQ, East Lansing, MI, USA) as previously described (Kuhlgert *et* *al*., 2016). For each analysis mean ± SD of four biological replicates was calculated.

### Thylakoid preparation and immunoblotting

For the OX2 line plus control, four separated pools of leaves, chosen with the same criteria used for pigment analysis, were analyzed. Thylakoid membranes were isolated and fractionated as previously described (Pinnola *et* *al*., 2015). Proteins were separated by sodium dodecyl sulfate polyacrylamide gel electrophoresis (SDS‐PAGE) in a Tris‐Tricine buffer system (Schägger & von Jagow, 1987). Immunotitration was performed as described by Cazzaniga *et* *al*. (2020) with antibodies (Agrisera, Vännäs, Sweden) against the proteins CP47 (AS11 1787), PSAA (AS06 172) LHCII (AS01 004) and AtpC (AS08312). For each analysis mean ± SD of four biological replicates was calculated.

### Chloroplast distribution

Protoplast images were captured using a Leica (Wetzlar, Germany) DM2500 microscope equipped with a DFC7000T camera. The distribution of chloroplasts was analysed using Imagej software (https://imagej.nih.gov/ij/download.html) on 70 images for each of the three transgenic lines and the control.

### Plant NAA treatment

*In* *vitro* cv Syrah stems of OX2 line and control, consisting of two apical leaves, one fully expanded and the other newly developing, were transferred to ½ Murashige & Skoog medium without or with two different 1‐naphthaleneacetic acid (NAA) concentrations (5 and 20 mg l^−1^). For each treatment, four stems of OX2 line and four stems of control were maintained in a growth chamber at 25°C with a 16 h : 8 h, light : dark photoperiod. Phenotype was observed 14 d after hormone treatment.

### Quantitative real‐time polymerase chain reaction (RT‐qPCR)

For transient expression, RNA was extracted from a pool of two apical well‐expanded leaves from each plant. For OXNAC33 and EARNAC33 transgenic plants, RNA was extracted from leaves collected and pooled following the same criteria used for pigment analysis. Total RNA was isolated from 50–100 mg of ground material using the Spectrum Plant Total RNA kit (Merck KGaA, Darmstadt, Germany). Gene expression analysis by qPCR was performed as previously described (Zenoni *et* *al*., 2011) using the primer sequences listed in Table S1. Each value corresponds to the mean ± SD of three technical replicates relative to the *VviUBIQUITIN1* (VIT_16s0098g01190) control.

### Transcriptomic analysis

The microarray analysis was performed with the RNA isolated for qPCR. For transient expression, the four most highly overexpressing plants (nos. 3, 5, 6 and 7) were selected and used as biological replicates, while for OXNAC33 transgenic lines, the OX1, 2 and 3 lines were used as biological replicates. The cDNA synthesis, labelling, hybridization and washing were performed according to the Agilent Microarray‐Based Gene Expression Analysis Guide (v.6.5). Each sample was hybridized to an Agilent custom microarray four‐pack 44K format (Agilent Sure Print HD 4X44K 60‐mer; cat. no. G2514F‐048771; Dal Santo *et* *al*., 2016) and scanned using an Agilent Scanner (G2565CA; Agilent Technologies, Santa Clara, CA, USA). Feature extraction and statistical analysis of the microarray data was conducted as reported by Amato *et* *al*. (2017). Differentially expressed genes (DEGs) were identified by Student’s *t* test (α = 0.05), assuming equal variance among samples, and selected by fold change |1.5|).

### Dual luciferase assay

The *VviNAC33* (1320 bp), *SGR1* (1558 bp), *ATG8f* (1616 bp), *RopGEF1* (1604 bp), *PIN1* (1637 bp) and *ATPC1* (1573 bp) promoter regions were amplified by PCR from cv Corvina genomic DNA using KAPA HiFi DNA polymerase. The cloning steps and Dual Luciferase Reporter Assay were carried out as described by Amato *et* *al*. (2019), using as effector vectors *35S: VviNAC33* with *35S: VviWRKY19* (VIT_07s0005g01710) and *35S: VvibHLH75* (VIT_17s0000g00430), both previously reported by Fasoli *et* *al*. (2018). Mean ± SD of four biological replicates was calculated.

## Results

### *VviNAC33* is upregulated during the vegetative‐to‐mature transition of several organs and is strongly expressed in leaves undergoing senescence

**Fig. 1:**
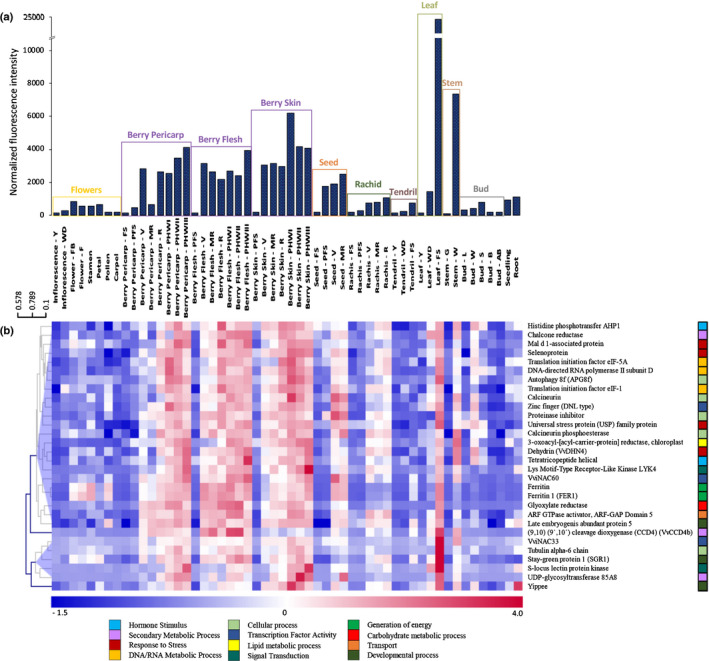
*VviNAC33* expression analysis in grapevine organs. (a) *VviNAC33* expression profile in *Vitis vinifera* cv Corvina in 54 grape organs during development (Fasoli *et al*., 2012). (b) Heat map for the set of 30 genes closely co‐expressed with *VviNAC33*. This core set was defined using *VviNAC33* as bait in the Corvina atlas (Supporting Information Dataset S1). The heat map shows the expression profiles of the 30 genes during grapevine organ development. Clusters were generated by hierarchical clustering in Tmev software, considering the expression value of each gene in different organs. Bud‐AB, bud after burst; Bud‐B, bud burst; Bud‐L, latent bud; Bud‐S, bud swell; Bud‐W, winter bud; Flower‐F, flowering; Flower‐FB, flowering begins; FS, fruitset; Inflorescence‐WD, well‐developed; Inflorescence‐Y, young; Leaf‐FS, senescing leaf; Leaf‐WD, mature; Leaf‐Y, young; MR, mid‐ripening; PFS, post‐fruitset; R, ripening; Stem‐G, green; Stem‐W, woody; Tendril‐FS, mature; Tendril‐WD, well‐developed; Tendril‐Y, young; V, veraison.

The analysis of previously released transcriptomic databases (Fasoli *et* *al*., 2012) revealed that *VviNAC33* is expressed at high levels during senescence in leaves and in woody stems, but only weakly expressed in the young leaf, green stem, flower organs, pollen, bud and tendrils. *VviNAC33* expression is also induced before veraison in developing seeds and at veraison in berries (Fig. 1a), and a similar sharp increase was observed during berry softening in different grapevine genotypes and during the 10 d before veraison (Fig. S1a,b; Massonnet *et* *al*., 2017; Fasoli *et* *al*., 2018). Moreover, the *VviNAC33* expression profile during berry development belonged to a stage‐specific cluster (Dal Santo *et* *al*., 2018), and was thus unaffected by environmental conditions or G × E interactions (Fig. S1c,d).

Co‐expression analysis using a grapevine expression atlas (Fasoli *et* *al*., 2012) revealed 92 genes that were positively correlated to *VviNAC33*, given a Spearman coefficient > 0.7 (Dataset S1). The top‐30 most strongly co‐expressed genes with putative functional annotations formed two clusters based on their expression profiles during organ development. The first was characterized by a sharp increase in expression during berry and seed development, similar to that observed during leaf senescence (Fig. 1b; upper cluster). This large cluster included another NAC member, *VviNAC60*, and genes involved in stress responses and cellular processes, such as *AUTOPHAGY* *8f* (*ATG8f*). The second was characterized by a stronger induction of expression during senescence (Fig. 1b; lower cluster). This smaller cluster included the *α6 TUBULIN CHAIN* gene, the *SENESCENCE‐INDUCIBLE* *CHLOROPLAST* *STAY‐GREEN* *PROTEIN* *1* (*SGR1*), and genes encoding the S‐locus lectin protein kinase and the UDP‐glycosyltransferase 85A8.

### *VviNAC33* belongs to the same clade as *ANAC092/ORE1*, a key activator of leaf senescence

The full‐length coding region of *VviNAC33* was amplified from cv Corvina berry cDNA. The 888‐bp open reading frame encodes a protein of 296 amino acids with a predicted mass of 33.38 kDa and a calculated isoelectric point (pI) of 6.33 (Fig. S2). A phylogenetic tree was constructed for *VviNAC33* and the other 73 known grapevine NAC genes (Wang *et* *al*., 2013) whose sequences were downloaded from the new VCost.v3 genome annotation (Canaguier *et* *al*., 2017) (Table S2). The phylogenetic tree revealed some differences from the previous phylogenetic analysis (Wang *et* *al*., 2013), both in terms of clade designation and gene distribution among clades. *VviNAC33* belongs to clade V and is closely related to *VviNAC56* and *VviNAC14* (Fig. S3). However, the three NAC genes showed different expression profiles, with *VviNAC33* abundantly expressed in leaves undergoing senescence, *VviNAC56* strongly expressed in stamens, and *VviNAC14* expressed in buds (Fig. S4).

**Fig. 2:**
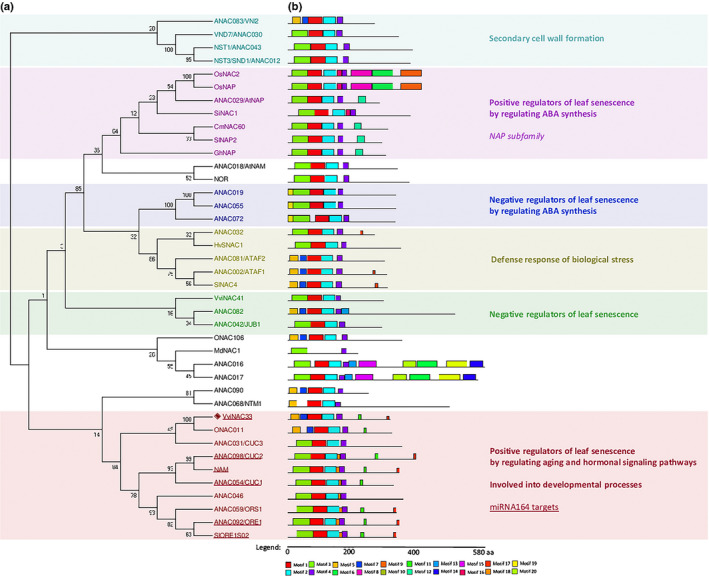
Phylogenetic relationships and motif compositions of VviNAC33 and 39 additional NAC protein sequences. (a) Phylogenetic tree (neighbour‐joining method) of VviNAC33 and 36 additional NAC protein sequences with functional annotations from various species prepared in mega7 (Kumar *et al*., 2016). The numbers next to the nodes are bootstrap values from 1000 replicates. VviNAC33 is labelled with a red rhombus. The following GenBank accession nos. were used: *Arabidopsis thaliana* ATAF1/ANAC002 (AT1G01720), ATAF2 (AT5G08790), ANAC016 (AT1G34180), ANAC017 (AT1G34190), ANAC019 (AT1G52890), ANAC032 (AT1G77450), ANAC046 (AT3G04060), ANAC055 (AT3G15500), ANAC072 (AT4G27410), ANAC082 (AT5G09330), ANAC090 (AT5G22380), AtNAM (AT1G52880), AtNAP/ANAC029 (AT1G69490), CUC1 (AT3G15170), CUC2 (AT5G53950), CUC3 (AT1G76420), JUB1/ANAC042 (AT2G43000), ORE1/ANAC092 (AT5G39610), ORS1/ANAC059 (AT3G29035), NTM1 (AT4G01540), NST1/ANAC043 (AT2G46770); NST3/SND1/ANAC012 (AT1G32770); VND7/ANAC030 (AT1G71930); VNI2/ANAC083 (AT5G13180); *Cucumis melo* CmNAC60 (XM_008448163); *Hordeum vulgare* HvSNAC1 (AEG21060.1); *Gossypium hirsutum* GhNAP (ALG62640.1); *Oryza sativa* ONAC011 (Os06g0675600), ONAC106 (Os08g0433500), OsNAC2 (Os03g0327800), OsNAP (Os03g0327800); *Malus domestica* MdNAC1 (MF401514.1); *Petunia hybrida* NAM (CAA63101); *Setaria italica* SiNAC1 (XP_004967928.1); *Solanum lycopersicum* NOR (Solyc10g006880), SlNAC4 (Solyc11g017470), SlNAP2 (Solyc04g005610.2.1), SlORE1S02 (Solyc02g088180). (b) Schematic representation of motifs in the same set of NAC proteins identified by meme analysis. Each color represents a specific motif. See also sequence logo in Supporting Information Fig. S5.

In order to explore the role of VviNAC33, a phylogenetic tree was constructed with VviNAC33 and 39 additional NAC proteins that have been functionally characterized in different plant species (Fig. 2a). The closest matches to VviNAC33 were ONAC011 in rice, a positive regulator of heading and senescence (El Mannai *et* *al*., 2017), and the Arabidopsis protein CUP SHAPED COTYLEDON 3 (CUC3), involved in shoot meristem formation (Vroemen *et* *al*., 2003). The Arabidopsis CUC1 and CUC2 and the *Petunia* *hybrida* protein NAM, which are all involved in initiating the shoot apical meristem and establishing organ boundaries (Souer *et* *al*., 1996; Raman *et* *al*., 2008), also belong to the same clade as VviNAC33, along with the proteins ANAC092/ORE1, a key positive regulator of leaf senescence in Arabidopsis (Kim *et* *al*., 2016; Xi *et* *al*., 2019), and SlORE1S02 in tomato (Lira *et* *al*., 2017). Interestingly, several members of the VviNAC33 cluster are targets of *miR164* (Fig. 2; Sun *et* *al*., 2012), supporting a similar transcriptional regulation of these NACs.

The predicted sequence of the VviNAC33 protein (Fig. S5) contains seven motifs, including two conserved C‐terminal motifs shared only with proteins of the VviNAC33 clade (Fig. 2b), and a monopartite N‐terminal nuclear localization signal (PKGKKTD) (Fig. S2).

### VviNAC33 localizes to the nucleus and binds to a conserved NAC motif

**Fig. 3:**
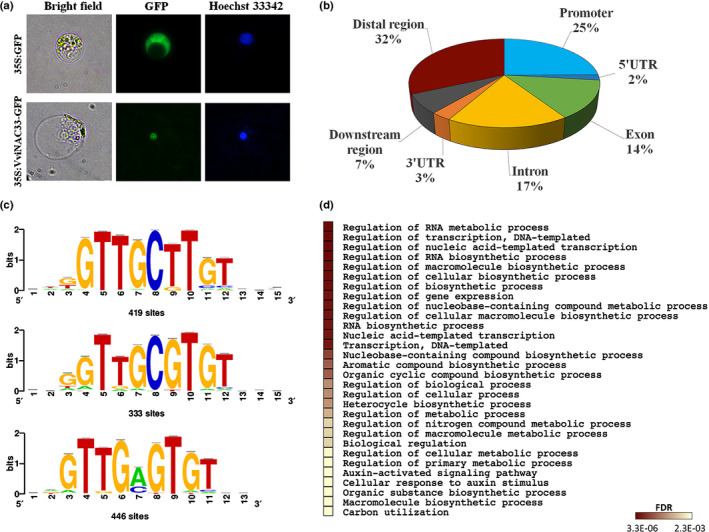
VviNAC33 subcellular localization and DAP‐seq results. (a) Subcellular localization of VviNAC33. Grapevine protoplasts isolated from Corvina leaves were transformed with the *35S:GFP* and *35S:VviNAC33‐GFP* constructs. (b) Distribution of VviNAC33 binding sites. (c) VviNAC33 binding to three core motifs identified by RSAT Plants. (d) GO enrichment analysis on 971 putative VviNAC33 targets. The heatmap indicates the 20 most significant pathways (*P*‐value cut‐off, false discovery rate (FDR) ≤ 0,01). Enrichment analysis was based on hypergeometric distribution followed by FDR correction.

The subcellular localization of VviNAC33 was assessed through protoplast transformation with the construct *35S:VviNAC33‐GFP* or the control vector *35S:GFP*. In protoplasts transformed with the control vector, GFP fluorescence was detected in both the nucleus and cytosol, but in protoplasts transformed with *35S:VviNAC33‐GFP* the GFP fluorescence was concentrated in the nucleus and co‐localized with the nuclear stain Hoechst 33342 (Fig. 3a).

Next, we carried out DAP‐seq assays to identify the putative direct targets of VviNAC33. We initially observed 4427 enriched peaks (Dataset S2), which was reduced to 3910 by removing those with a sample/control ratio < 5 (Dataset S3). The distribution of peaks revealed that 25% were located within promoter regions (up to 2 kb upstream from a transcription start site), 2% were located in 5′ untranslated regions (UTRs), 14% were located in exons, 17% were located within introns, 3% were located in 3′ UTRs, 7% were located in the 3‐kb downstream region, and 32% were intergenic (Fig. 3b), leading to 3457 candidate target genes.

We identified three major binding motifs (GTTG(C/A)(T/G)TGT) with strong significance (Fig. 3c) and phylogenetic footprints correlating with ANAC092/ORE1 (Fig. S6a). Distribution analysis revealed that 1024 peaks (27% of the total) were in the upstream region (promoter or 5′ UTR) of 971 genes, highlighting potential VviNAC33 direct targets. We focused our analysis on this specific set and found three slightly different top‐ranking binding motifs, but the correlation with ANAC092/ORE1 was maintained (Fig. S6b). We found three top VviNAC33 dimeric binding motifs, confirming that the well‐known characteristic of NAC TFs to bind DNA as stable homodimers or heterodimers is preserved in grapevine (Fig. S6b). Gene ontology (GO) enrichment analysis of the 971 putative direct target genes revealed a significant overrepresentation (FDR < 0.01) of genes in categories including ‘Regulation of biosynthetic process’, ‘Regulation of transcription’, ‘Regulation of gene expression’, ‘Auxin‐activated signaling pathway’ and ‘Cellular response to auxin stimulus’ (Fig. 3d).

By ranking the putative targets by *P*‐value, we found that the top position was occupied by the photosystem II (PSII) gene *PsbA*, followed by the regulator of Chl degradation *SGR1* (Dataset S3). Interestingly, the 971 putative VviNAC33 targets included 14 switch genes from the 113 identified in the atlas dataset (Fasoli *et* *al*., 2012), 17 of the 225 berry‐specific switch genes reported by Massonnet *et* *al*. (2017) and several markers of the transcriptional transition characterizing the onset of berry ripening (Dataset S3).

### Transient overexpression of VviNAC33 identifies positively and negatively regulated targets

**Fig. 4:**
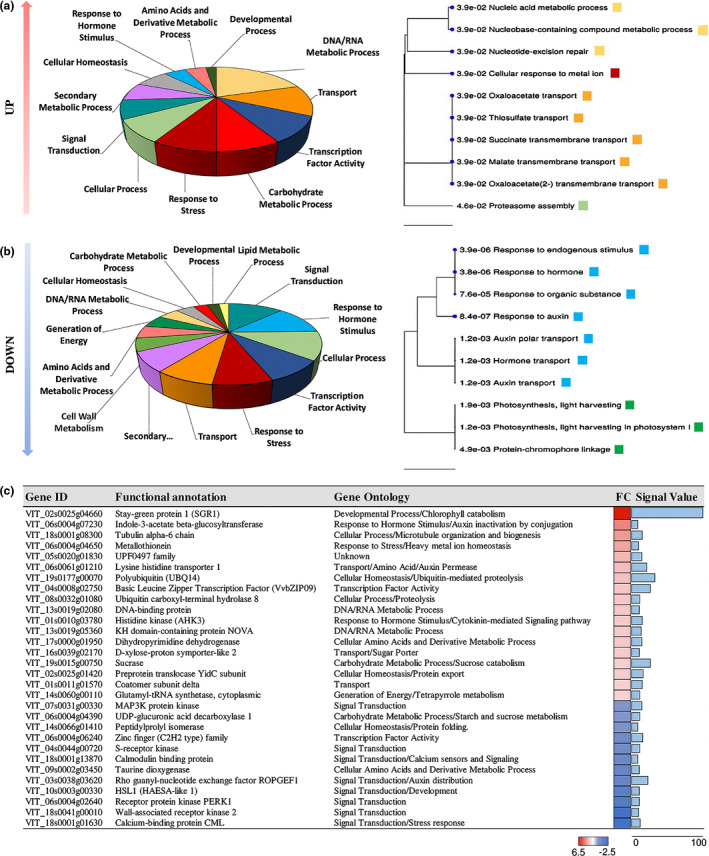
Transcriptomic analysis of grapevine leaves in transient expression experiments. (a) Gene ontology (GO) distribution (left) and GO enrichment (right) analysis of the 122 upregulated genes in leaves transiently overexpressing *VviNAC33* (threshold |FC| > 1.5.) (b) Gene ontology distribution (left) and GO enrichment (right) analysis of 257 downregulated genes in in leaves transiently overexpressing *VviNAC33* (|FC| > 1.5). The hierarchical clustering trees summarize correlations among the 10 most significant pathways (false discovery rate (FDR) ≤ 0.05). Pathways with many shared genes are clustered together. Bigger dots indicate more significant *P*‐values. (c) Common genes in the list of differentially expressed genes (DEGs) identified by transient expression and the list of putative VviNAC33 targets based on DNA affinity purification sequencing (DAP‐seq) analysis. Only the 33 functionally annotated genes are reported. FC, fold change.

To investigate the ability of VviNAC33 to activate or inhibit gene expression, we infiltrated grapevine plantlets (cv Sultana) with *A*. *tumefaciens* (Amato *et* *al*., 2017) carrying the *35S: VviNAC33* overexpression construct. The infiltrated plants did not show any overt phenotype, so we used RT‐qPCR to quantify transgene expression and selected four lines strongly overexpressing *VviNAC33* for transcriptomic analysis (Fig. S7). We identified 1027 DEGs based on a *t*‐test with a significance threshold of *P* < 0.05 (Dataset S4), 379 of which satisfied the fold‐change criterion |FC| > 1.5. This comprised 122 upregulated and 257 downregulated genes. Gene ontology (GO) distribution and enrichment analysis showed that the upregulated genes mainly represented the functional categories ‘DNA/RNA metabolic process’, ‘Transport’ and ‘Cellular response to ions’ (Fig. 4a), whereas the downregulated genes mainly represented the functional categories ‘Response to hormone stimulus’, ‘Auxin transport and signaling’ and ‘Photosynthesis’ (Fig. 4b). Interestingly, some of the most strongly upregulated DEGs were co‐expressed with *VviNAC33*, including *SGR1*, *IAGLU* (*indole‐3‐acetate β‐glucosyltransferase*), *VvCCD4b*, encoding a (9,10) (9′,10′) cleavage dioxygenase, and the *α6 TUBULIN CHAIN* gene (Dataset S4).

By aligning the 1027 DEGs with the 971 putative VviNAC33 targets determined by DAP‐seq analysis, we identified 40 genes common to both lists, 24 of which were upregulated and 16 downregulated, suggesting VviNAC33 acts directly as both an activator and repressor (Fig. 4c). *SGR1* was the most strongly upregulated direct target candidate with the highest DAP‐seq signal, with *IAGLU* and the *α6 TUBULIN CHAIN* gene also ranking highly. Downregulated putative targets included several genes encoding signaling components such as *RopGEF1*, an activator of plant Rho GTPases (ROPs), which are involved in the polar distribution of auxin influx carrier AUX1 and the accumulation of PIN efflux carriers (Liu *et* *al*., 2017).

### The stable overexpression of *VviNAC33* induces leaf de‐greening by modulating photosynthetic complexes and leaf expansion

The preliminary functional analysis of *VviNAC33* in *N*. *benthamiana* leaves by transient expression revealed the acceleration of leaf de‐greening compared to vector controls, confirmed by a significant reduction in Chl amount, Chl : carotenoid ratio and maximum quantum efficiency (*F*_v_/*F*_m_) of PSII (Fig. S8a–d).

We therefore generated transgenic grapevine plants expressing *VviNAC33* under the control of the *CaMV* *35S* promoter (OXNAC33 lines). We recovered 40 PCR‐positive OXNAC33 plantlets and 10 vector controls (data not shown). Ten of the OXNAC33 transgenic lines expressed *VviNAC33* at significantly higher levels than the control lines, and we observed a positive correlation between phenotypic alterations and the level of transgene expression (Fig. S9a,b). Three independent lines with the highest *VviNAC33* expression (nos. 4, 5 and 8) were selected for further analysis and renamed OX1, OX2, OX3 for simplicity. Southern blot analysis revealed that all three lines as well as the vector control contained a single‐copy transgene (Fig. S9c).

**Fig. 5:**
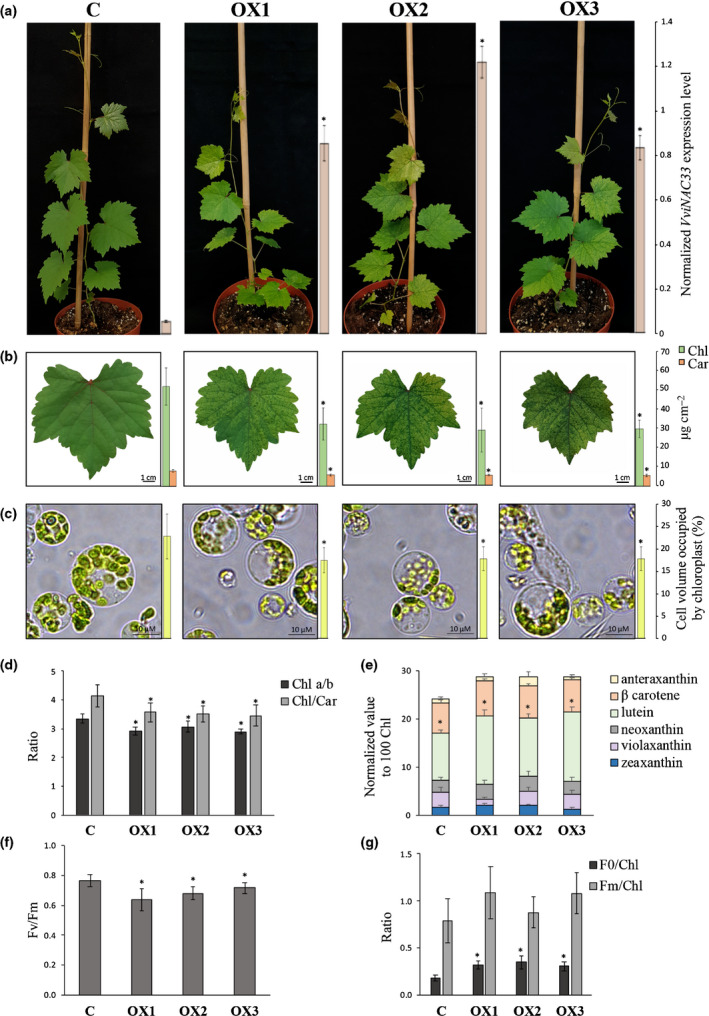
Phenotypic changes in transgenic grapevine plants overexpressing *VviNAC33*. (a) Whole‐plant phenotype caused by the stable expression of *VviNAC33* in selected OXNAC33 lines compared to vector controls. The expression levels detected by quantitative polymerase chain reaction (qPCR) are indicated by the bars next to the pictures. Each value corresponds to the mean ± SD of three technical replicates relative to the *VviUBIQUITIN1* (VIT_16s0098g01190) control. (b) Fully expanded leaves showing the phenotype caused by the stable overexpression of *VviNAC33* in the three OXNAC33 lines compared to the vector control. Total Chl and carotenoid (‘Car’) levels are indicated by the bars next to the pictures. (c) Distribution of chloroplasts in protoplasts isolated from the three OXNAC33 lines compared to the vector control. The proportion of cell volume occupied by chloroplasts, as determined by light microscopy, is indicated by the bars next to the pictures. (d) Chl*a* : Chl*b* and Chl : carotenoid (Chl : Car) ratios. (e) Carotenoid content, with individual carotenoid values normalized to 100 Chl equivalents. (f) *F*_0_ and *F*_m_ normalized to Chl. (g) Photosystem II maximum quantum efficiency (*F*_v_/*F*_m_). All pigment and photosynthetic performance data are expressed as mean ± SD (*n* = 4). Asterisks (*) indicate significant differences (*, *P* < 0.05; *t*‐test) in the OXNAC33 lines compared to the vector control. *F*_0_, minimal fluorescence from dark‐adapted leaf; *F*_m_, maximal fluorescence from dark‐adapted leaf.

The growth rate, height and general habitus of the OXNAC33 transgenic lines were similar to the controls (Fig. 5a). However, as described for *N*. *benthamiana*, mature leaves exhibited a clearly visible de‐greening effect leading to a green/pale green variegated phenotype that became more apparent with age. Pigment analysis revealed a sharp reduction (*c*. 40%) in the concentrations of Chl and carotenoids (Fig. 5b). The leaf area of the transgenic plants was *c*. 35% lower than the controls (Fig. S9d). We isolated protoplasts from transgenic and control leaves and found a significant (*c*. 20%) reduction in the cell area occupied by chloroplasts in the transgenic lines, which only partially explained the loss of pigments (Fig. 5c). We therefore measured the Chl*a* : Chl*b* and Chl : carotenoid ratios, both of which were lower in the OXNAC33 transgenic lines. More detailed analysis of the carotenoid content revealed that the lower Chl : carotenoid ratio in the transgenic plants was almost entirely due to the higher concentrations of lutein. The analysis of the fluorescence yield in dark‐adapted leaves revealed a significant decrease in *F*_v_/*F*_m_ in the three transgenic lines. Whereas maximal fluorescence from dark‐adapted leaf (*F*_m_) (normalized to the Chl content) remained stable, minimal fluorescence from dark‐adapted leaf (*F*_0_) was significantly higher in the transgenic leaves (Fig. 5d–g).

**Fig. 6:**
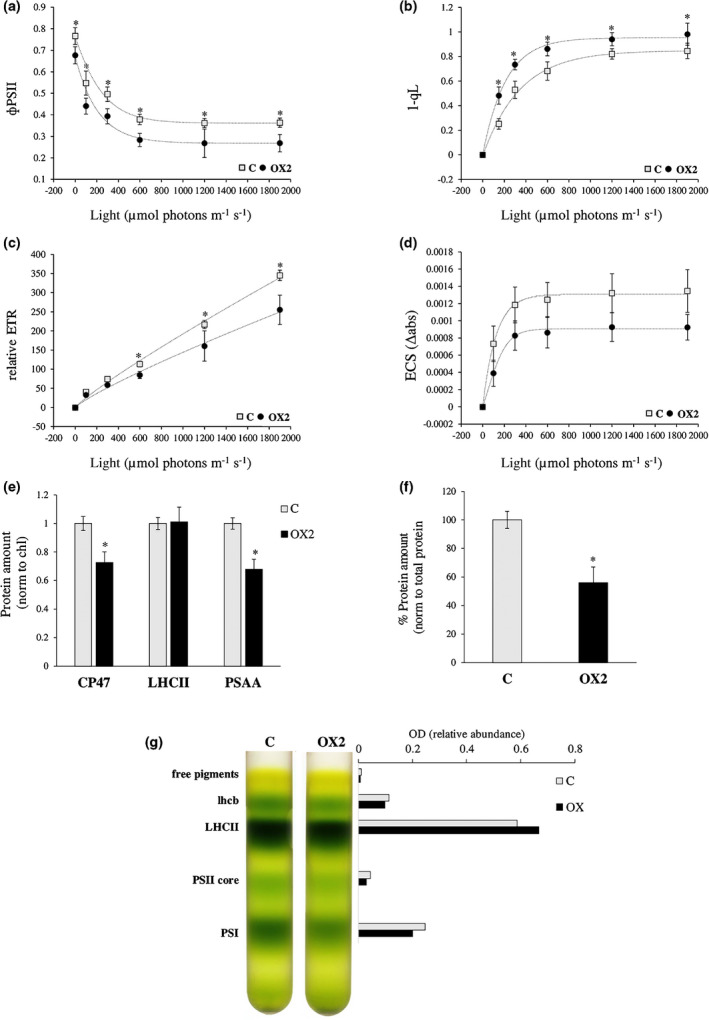
Photosynthetic parameters and organization of thylakoid membranes. (a) Dependence of photosystem II operating efficiency (ΦPSII), (b) 1‐qL (estimates the fraction of PSII centers with reduced first quinone acceptor (QA)), (c) electron transport rate (ETR) and (d) total proton motive force (ECS) on actinic light intensity for the OX2 line compared to the vector control. (e) Immunotitration of thylakoid proteins using specific antibodies against PSAA, LHCII and PSAA corrected for Chl content, and (f) ATPC1 corrected for total protein content. (g) Sucrose density gradient fractionation. The composition of the green bands is shown on the left, and the relative abundance of the band (normalized to total green content on the gradient) is shown on the right. All data are expressed as mean ± SD (*n* = 4). Asterisks (*) indicate significant differences (*, *P* < 0.05; *t*‐test) between the OX2 line and the vector control.

We selected line OX2, which expressed *VviNAC33* at the highest level, for the analysis of photosynthetic parameters in more detail at different light intensities. The PSII operating efficiency (ΦPSII), electron transport rate (ETR) and electrochemical proton gradient (determined by ECS) were significantly lower in OX2 compared to the control at all light intensities, whereas the 1‐qL parameter representing the redox state of the plastoquinone pool was higher in OX2, suggesting the plastoquinone pool was more reduced by electrons (Fig. 6a–d). Nonphotochemical quenching (NPQ) was enhanced in the OX2 leaves at higher light intensities (Fig. S10a). Finally, dark‐adapted leaves were exposed to saturating actinic light, and the OJIP curve was registered in order to monitor the polyphasic rise from *F*_0_ to *F*_m_ (Stirbet *et* *al*., 2018). The steeper O–J part of the curve (Fig. S10b) indicates lower electron‐trapping efficiency in the OX2 leaf and thus an increase in the dissipation of absorbed light energy by fluorescence (Stirbet & Govindjee, 2011).

We then measured the relative amounts of photosynthetic components by immunoblotting on isolated thylakoid membranes. We found that CP47 (PSII core), PSAA (PSI core) and ATPC1 were significantly depleted in OX2 leaves compared to controls, whereas LHCII was present at similar levels. We also fractionated the native Chl binding complexes on a sucrose gradient following thylakoid solubilization. The analysis of free pigments, monomeric LHCB, trimeric LHCII, PSII core and PSI‐LHCI confirmed a relative increase in the content of antenna subunits, particularly LHCII, and a relative decrease in the PSII and PSI fractions in OX2 compared to the control (Fig. 6e–g).

### Transcriptomic analysis of transgenic leaves reveals additional putative direct targets of VviNAC33

**Fig. 7:**
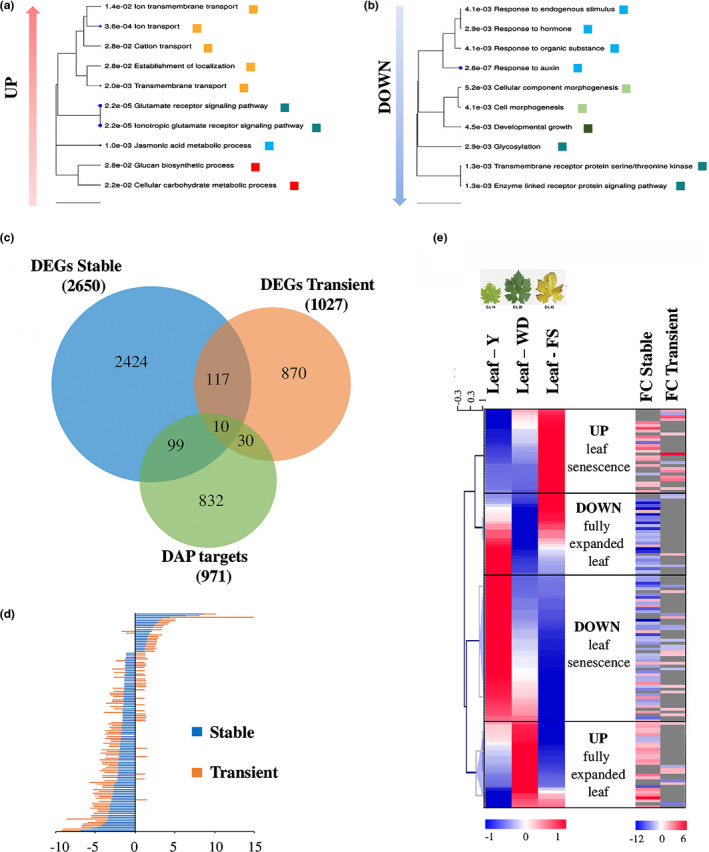
Transcriptomic analysis of transgenic grapevine plants overexpressing *VviNAC33*. (a) Gene ontology (GO) enrichment analysis of the upregulated genes in transgenic leaves with |FC| > 1.5. (b) Gene ontology enrichment analysis of the downregulated genes in transgenic leaves with |FC| > 1.5. (c) Venn diagram showing genes common to the lists of differentially expressed genes (DEGs) in the transgenic plants and transient expression experiments, and to the targets identified by DNA affinity purification sequencing (DAP‐seq) analysis. (d) Histogram showing the fold changes (FC) in expression of the genes classed as DEGs by both transient expression experiments and transgenic plants. (e) Heat map of the 139 putative direct targets of VviNAC33 in three leaf developmental stages (Fasoli *et al*., 2012). Clusters were generated by hierarchical clustering in Tmev, considering the expression value of each gene in the different stages (left) and the FC of the 139 putative targets. Boxes indicated four expression trends: the upregulated and downregulated genes at senescence and in the fully expanded leaves.

We analyzed leaves of OXNAC33 transgenic lines and controls for genes that are differentially expressed before the phenotype appears. In total, we identified 2650 DEGs (*t*‐test, *P* < 0.05), 617 of which were upregulated and 1388 downregulated with a |FC| > 1.5 (Dataset S5). Gene ontology (GO) enrichment analysis showed that ‘Glutamate signaling pathway’, ‘Transport’ and ‘Carbohydrate metabolic process’ were the most significant overrepresented functional categories for the upregulated genes, whereas ‘Response to auxin’, ‘Developmental growth’, ‘Cell morphogenesis’ and ‘Signaling pathway’ were the most significant overrepresented functional categories for the downregulated genes (Fig. 7a), in agreement with our transient expression experiments (Fig. 4a,b).

We identified 127 common DEGs among the 2650 identified in transgenic plants and the 1027 identified by transient expression (Fig. 7b,c; Table S3).

By combining the DEGs identified in the transgenic plants and transient expression experiments with the DAP‐seq data, we assembled a list of 139 genes that are most likely to be direct targets of VviNAC33 (Fig. 7b; Table S4). All 139 genes were screened against the three leaf development stages included in the transcriptomic atlas, revealing four major expression trends: upregulation at senescence, downregulation at senescence, peak of expression in the mature leaf, and trough of expression in the mature leaf. There was consistency between the behavior of these genes in the expression atlas and transgenic plants, with those induced by VviNAC33 tending to be upregulated at senescence or in the mature leaf and those suppressed by VviNAC33 tending to be downregulated at senescence or in the mature leaf (Fig. 7d; Table S4). Interestingly, most of the DEGs identified by transient expression corresponded to those modulated at senescence, indicating that transient expression highlights the impact of VviNAC33 at its highest expression level. Genes strongly expressed at senescence included *SGR1*, *IAGLU* and the *α6 TUBULIN CHAIN* gene (Table S4).

Putative target genes upregulated by stable *VviNAC33* overexpression included several involved in nutrient transport, metabolism and recycling, such as genes encoding a substrate carrier, d‐xylose proton symporter‐like 2, sucrase, and ATG8f (Table S4). On the other hand, putative targets downregulated by stable *VviNAC33* overexpression included several genes involved in auxin signaling, such as *RopGEF1* and *PIN1*, as well as *ATPC1*, encoding the ATP synthase γ chain 1 t which is involved in PSII integrity and activity.

In addition to putative direct targets, in OXNAC33 transgenic lines we found several downregulated genes involved in the synthesis of Chl and carotenoids, in the spatial regulation of chloroplast division and in auxin signaling (Dataset S5).

Considering several clues about the possible role of VviNAC33 in controlling auxin signaling and metabolism emerged from DAP‐seq and transcriptomic analyses (Table S5), we tested whether exogenous auxin treatment was able to rescue the de‐greening phenotype in stable *VviNAC33* overexpressing plants. We treated *in* *vitro* shoots of the OX2 and control line with two NAA concentrations (Fig. S11). After 2 wk we found that both NAA concentrations were able to restore the green phenotype in newly formed leaves, which appeared similar to the controls, while the fully expanded leaves maintained the de‐greening effect (Fig. S11).

### A VviNAC33‐EAR chimeric repressor boosts the Chl content and growth of transgenic grapevine leaves

To create a dominant suppressor which overcomes the activity of endogenous VviNAC33, we fused the C‐terminal domain of VviNAC33 to the plant‐specific EAR repression domain (Ohta *et* *al*., 2001; Hiratsu *et* *al*., 2002) and placed the chimeric repressor under the control of the endogenous *VviNAC33* promoter. The chimeric repressor construct was used to stably transform embryogenic grapevine callus and produce transgenic vines. We recovered four PCR‐positive plantlets containing *VviNAC33‐EAR* and two containing the vector control (data not shown). Transgene expression was confirmed by RT‐qPCR in fully expanded leaves, when the expression of endogenous *VviNAC33* begins to increase. Three independent EARNAC33 lines (nos. 1, 2 and 4) were selected based on their strong transgene expression and were renamed EAR1, EAR2 and EAR3 for simplicity (Fig. S12a,b).

**Fig. 8:**
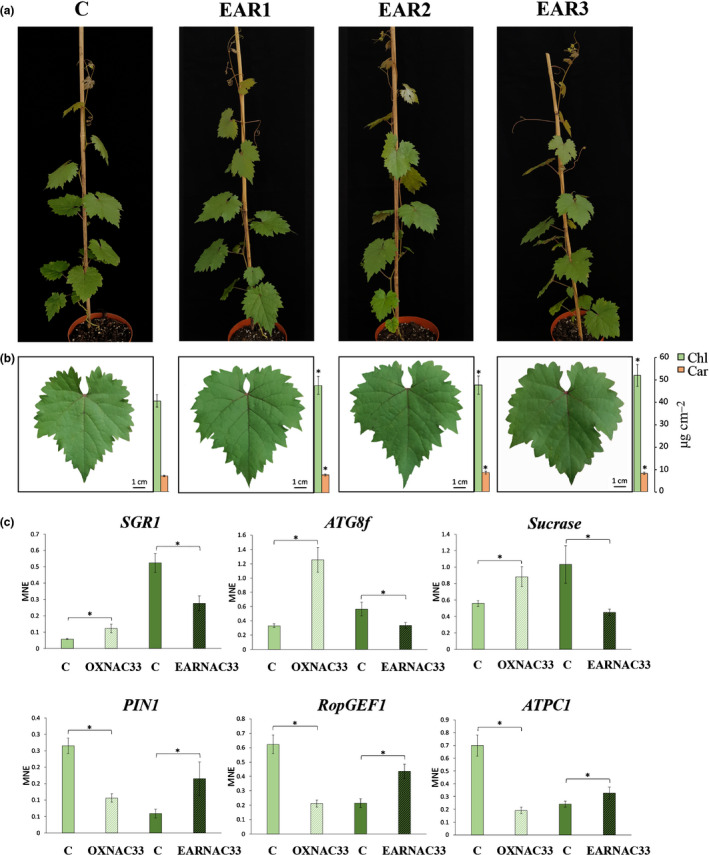
Phenotypic changes in transgenic grapevine plants expressing the repressor NAC33‐EAR and the expression level of *VviNAC33* targets. (a) Whole‐plant phenotype caused by the stable expression of *VviNAC33‐EAR* in the three EARNAC33 lines compared to the vector control. (b) Fully expanded leaf phenotype caused by the stable expression of *VviNAC33‐EAR* in the three EARNAC33 lines compared to the vector control. Total Chl and carotenoid (‘Car’) levels are indicated by the bars next to the pictures. Data are mean ± SD (*n* = 4). Asterisks indicate significant differences (*, *P* < 0.05; *t*‐test) between the EARNAC33 lines and vector control. (c) Expression levels of *SGR1*, *ATG8f*, *sucrase*, *PIN1*, *RopGEF1* and *ATPC1* in the leaves of transgenic plants overexpressing *VviNAC33* (OXNAC33) or expressing *VviNAC33‐EAR* (EARNAC33), determined by quantitative polymerase chain reaction (qPCR). Each value corresponds to the mean ± SD of three technical replicates relative to the *VviUBIQUITIN1* (VIT_16s0098g01190) control. Asterisks indicate significant differences (*, *P* < 0.05; *t*‐test) between the OXNAC33 or EARNAC33 lines and the vector control. MNE, mean expression level.

The three EARNAC33 lines showed normal vegetative growth, but the fully expanded leaves were significantly larger than controls (Figs 8a,b, S12c). Pigment content analysis revealed a significant increase in the Chl and carotenoid content of the transgenic lines (Fig. 8b). However, the Chl*a* : Chl*b* and Chl : carotenoid ratios, *F*_v_/*F*_m_, *F*_0_/Chl and *F*_m_/Chl were similar in transgenic plants and vector controls (Fig. S13a–c), possibly reflecting the partial but not complete dominance of the chimeric repressor.

The chimeric repressor system was also used to evaluate the expression of six putative VviNAC33 direct target genes by qPCR analysis, comparing transgenic lines OX2 and EAR2 and their controls. We assessed the upregulated candidate genes *SGR1*, the *sucrase* gene, and *ATG8f* and the downregulated candidate genes *PIN1*, *RopGEF1* and *ATPC1*. We found that *SGR1*, *ATG8f* and the *sucrase* gene were strongly upregulated by VviNAC33 but inhibited by VviNAC33‐EAR; conversely, *PIN1*, *RopGEF1* and *ATPC1* were strongly downregulated by VviNAC33 but not inhibited by VviNAC33‐EAR (Fig. 8c). This indicates that the addition of the EAR motif could have impaired the VviNAC33 repressor activity, possibly by destabilizing putative interactions with binding partners involved in repression or by inhibiting the expression of a repressor that is itself transcriptionally activated by VviNAC33.

**Fig. 9:**
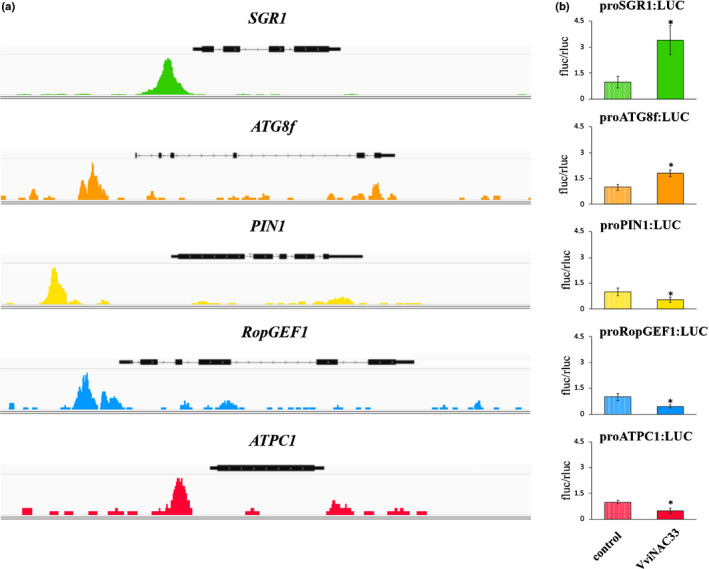
VviNAC33 binding sites and regulation of *SGR1*, *ATG8f*, *PIN1*, *RopGEF1* and *ATPC1*. (a) Integrative genomics viewer (IGV) images of the VviNAC33 DAP‐seq reads mapping to the promoters of the grapevine *SGR1*, *ATG8f*, *PIN1*, *RopGEF1* and *ATPC1* genes and (b) promoter activation tested by dual‐luciferase reporter assays in infiltrated *Nicotiana benthamiana* leaves. The activity of these promoters was tested in the presence and absence of the *35S:VviNAC33* effector vector. LUC values are reported relative to the REN value and normalized against the control (empty effector vector). LUC values represent the mean of four biological replicates ± SD. Asterisks indicate significant differences in promoter activation compared with the control (*, *P* < 0.01; *t*‐test).

We then tested the ability of VviNAC33 to directly induce or repress the expression of the putative target genes validated by qPCR by a dual‐luciferase assay in *N*. *benthamiana* leaves. The upstream regions of these genes containing NAC33 DAP‐seq peaks (Fig. 9a) were cloned upstream of a *35S* promoter‐luciferase cassette. Sequence analysis of these putative promoter regions showed several putative VviNAC33 binding sites, including a conserved motif near the summit of each DAP‐seq peak (Fig. S14). The dual‐luciferase assay showed that VviNAC33 significantly activates the *SGR1* and *ATG8f* promoters and represses the *PIN1*, *RopGEF1* and *ATPC1* promoters (Fig. 9b).

Finally, we investigate the ability of VvibHLH75 and VviWRKY19, two TFs previously suggested to regulate *VviNAC33* in grape berry (Fasoli *et* *al*., 2018), to activate the VviNAC33 expression. The analysis showed that *VviNAC33* is positively regulated by both VvibHLH75 and VviWRKY19 (Fig. S15).

## Discussion

In this work we characterized the function of VviNAC33 in regulating vegetative/green‐to‐mature/woody growth shift (Fasoli *et* *al*., 2012; Palumbo *et* *al*., 2014). By transgenic approaches we found that VviNAC33 induces organ de‐greening and suppresses vegetative growth. DNA affinity purification sequencing (DAP‐seq) and transcriptomic analyses provided evidence that 139 genes, bound by VviNAC33 in the upstream region, were differentially expressed in plants stably or transiently expressing VviNAC33, thus likely representing direct targets of regulation.

*SGR1* was one of the putative direct targets of VviNAC33 and was strongly co‐expressed with *VviNAC33* throughout grapevine organ development. We demonstrated that VviNAC33 directly activates *SGR1* expression, as previously shown for the Arabidopsis close homolog ANAC092/ORE1 (Qiu *et* *al*., 2015). SGR1 removes magnesium from Chl*a* as the first step in initiating its degradation in multiple species (Park *et* *al*., 2007; Sato *et* *al*., 2007; Hörtensteiner, 2009; Zhou *et* *al*., 2011). The phenotypes of OXNAC33 and EARNAC33 transgenic leaves showed that VviNAC33 induces leaf de‐greening by reducing the total Chl content. There was also a significant depletion of the PSI and PSII core relative to antenna subunits in OXNAC33 leaves. Given that Chl*b* is bound only by antenna proteins, whereas Chl*a* is bound by both core and antenna subunits, this result agrees with the lower Chl*a* : Chl*b* ratio in the OXNAC33 lines. In Arabidopsis, ectopic expression of *SGR1* in fully greened leaves reduces the abundance of Chl‐binding proteins in PSI/II and the light‐harvesting complex (LHC), indicating that SGR1 directly attacks the pigment–protein complexes, and the Chl‐depleted apoproteins may then be immediately degraded in the thylakoid membranes (Shimoda *et* *al*. 2016). The loss of pigment‐binding proteins in our OXNAC33 leaves supports this hypothesis.

We found that leaf de‐greening in our transgenic plants was, at least in part, associated with a smaller area occupied by chloroplasts. A similar phenotype was observed in Arabidopsis *glk1* *glk2* double mutants, which featured pale green leaves with mesophyll cells containing small chloroplasts with sparse thylakoid membranes that failed to form grana (Waters *et* *al*., 2009). GOLDEN2‐LIKE1 (GLK1) and GLK2 activate genes responsible for key steps in Chl biosynthesis, contributing to photosystem assembly and chloroplast development and maintenance (Waters *et* *al*., 2009).

The gene encoding the PSII central core reaction protein D1 (*PsbA*) showed the highest *P*‐value in our DAP‐seq data, and additional genes in this dataset included *LOW PSII ACCUMULATION 1* (*LPA1*) and *PHOTOSYSTEM ONE 3* (*APO3*), which are required for efficient PSII and PSI assembly (Dataset S3). Although the control of these putative targets by VviNAC33 requires confirmation, our results strongly indicate that VviNAC33 plays a key role in the assembly and stability of the photosynthetic apparatus. In this context, we also found that VviNAC33 negatively regulates the expression of *ATPC1*, which encodes the ATP synthase γ chain 1 t, which is involved in the production of ATP from ADP in the presence of a proton gradient across the membrane. Interestingly, the inactivation of this gene in Arabidopsis abolishes photophosphorylation and alters PSII activity by increasing NPQ and F_0_ while reducing *F*_v_/*F*_m_ (Dal Bosco *et* *al*., 2003). Our data clearly demonstrated that VviNAC33 directly activates *SGR1* expression and suppresses *ATPC1*, which combined with the identification of *PsbA* and other PSII component genes among putative VviNAC33 targets strongly indicates that VviNAC33 is directly involved in shutting down photosynthesis in greened organs that enter the vegetative‐to‐mature phase transition.

Chlorophyll degradation is an integral aspect of senescence, occurring during the final phase of development (Lim *et* *al*., 2007). Senescence involves a massive cellular proteolysis carried out by organelles known as autophagosomes, which form under the control of ATG proteins to break down damaged and superfluous proteins (Zhai *et* *al*., 2016; Fu *et* *al*., 2020). A role of VviNAC33 in the control of autophagy is suggested by the fact that one of the direct targets of VviNAC33 is *ATG8f*, and by the upregulation of the *ATG8d* and *ATG18d* genes (Mizushima & Komatsu, 2011; Su *et* *al*., 2020) in OXNAC33 leaves. Autophagy includes steps of ubiquitination (Mizushima *et* *al*., 1998; Ichimura *et* *al*., 2000) and delivery of autophagosomes to the vacuole, via the microtubule cytoskeleton. Interestingly, the polyubiquitin gene *UBQ14* and the *α6 TUBULIN CHAIN*, both of which are strongly induced during senescence, were identified among the VviNAC33 targets (Table S4). Our data suggest that VviNAC33 directly orchestrates the elimination of chloroplasts by controlling the expression of genes involved in autophagy and photosynthetic activity.

The leaves of the OXNAC33 plants were significantly smaller than controls, whereas those of the EARNAC33 plants were significantly larger, suggesting that VviNAC33 controls organ growth. In Arabidopsis plants overexpressing *ANAC092/ORE1*, the length of the root meristem differed from control plants and the primary roots were shorter, reflecting a slower rate of meristematic cell division based on the suppression of *PIN*, *YUCCA2* and *ARF* (Xi *et* *al*., 2019). Although we did not determine the cause of the OXNAC33 and EARNAC33 leaf phenotypes, we hypothesize that VviNAC33 and ANAC092/ORE1 play similar roles in the regulation of organ growth. Indeed, several auxin‐related genes were found among the 139 direct target candidates that were downregulated (Table S4). Furthermore, we demonstrated that VviNAC33 directly binds to the auxin influx carrier *PIN1* promoter, inhibiting its expression. Two other PIN genes were downregulated in OXNAC33 leaves, and *PIN8* has a VviNAC33‐binding site within an exon. We also found that the expression of *ARF3*, *ARF4*, *ARF6* and *ARF8* was modulated by VviNAC33 in the OXNAC33 lines, although we did not find VviNAC33 sites in their promoters. These data provide strong evidence that VviNAC33 is a negative regulator of the auxin pathway, thus suppressing cell division during leaf development.

We also demonstrated that *RopGEF1* is directly suppressed by VviNAC33. RopGEF1 maintains polar auxin transport by regulating the properties of auxin influx and efflux carriers such as AUX1 and PIN proteins, thereby influencing auxin‐dependent growth and development (Swarup & Péret, 2012; Liu *et* *al*., 2017). Interestingly, *AUX1* was downregulated in leaves overexpressing *VviNAC33*, supporting the role of VviNAC33 as a negative regulator of auxin transport and distribution by inhibiting *PIN1* and *RopGEF1*.

The *CRY1* gene encoding cryptochrome 1, a blue light photoreceptor, was also one of the putative VviNAC33 direct targets and was upregulated by the overexpression of *VviNAC33*. CRY1 negatively regulates the phytochrome‐interacting transcription factors PIF4 and PIF5, which in turn activate PIN genes (Ma *et* *al*., 2016; Pedmale *et* *al*., 2016; Boccaccini *et* *al*., 2020).

The ability of exogenous auxin treatment to rescue the de‐greening phenotype of *VviNAC33* overexpressing shoots further supports the idea that the senescence‐like phenotype of transgenic leaves could be due to an impairment of auxin signaling triggered by VviNAC33. Senescence is controlled by multiple hormones; ethylene, jasmonic acid, salicylic acid, abscisic acid and brassinosteroids act as inducers, while cytokinins, gibberellic acid and auxin act as inhibitors (Gan & Amasino, 1997). Given this complexity, the involvement of other hormones besides auxin in the leaf de‐greening phenotype and a role of VviNAC33 in their control, could not be ruled out.

We provided several lines of evidence showing that VviNAC33 is a key regulator of the shift toward organ senescence acting through molecular mechanisms similar to those previously described for the Arabidopsis protein ANAC092/ORE1 (Kim *et* *al*., 2006; Balazadeh *et* *al*., 2010). Both activate genes such as *SGR1* that trigger Chl degradation (Qiu *et* *al*., 2015) while repressing the expression of auxin transporter *PIN* genes to control growth (Xi *et* *al*., 2019). The *VviNAC33* and *ANAC092/ORE1* genes are also regulated in a similar manner, both being repressed by *miRNA164* (Kim *et* *al*., 2009; Sun *et* *al*., 2012). Interestingly, *miR164* is expressed strongly in young grapevine leaves but only weakly in old leaves and fruit, which is the inverse of the pattern of expression observed for *VviNAC33* (Belli Kullan *et* *al*., 2015).

There is strong evidence that some tomato NAC TFs involved in the control of leaf senescence are also involved in fruit ripening. SlORE1S02, SlORE1S03 and SlORE1S06 induce leaf senescence and source–sink sugar partitioning, affecting the final sugar concentration (Lira *et* *al*., 2017). Recently, NOR, a NAC TF that regulates fruit ripening in tomato, was shown to induce leaf senescence (Ma *et* *al*., 2019). While we could not determine the effects of VviNAC33 or VviNAC33‐EAR in transgenic berries because neither the transgenic plants nor vector controls flower under our growing conditions, a role for VviNAC33 at the onset of berry ripening is indicated by increased expression level over a few days before veraison.

Consistently, several genes previously identified as markers of the onset of berry ripening together with *VviNAC33* (Fasoli *et* *al*., 2018) were highlighted in this work among the DAP‐seq hits as potential direct targets of VviNAC33. In addition, we confirmed that the two berry ripening markers VvibHLH75 and VviWRKY19 are able to directly activate the expression of *VviNAC33*.

**Fig. 10:**
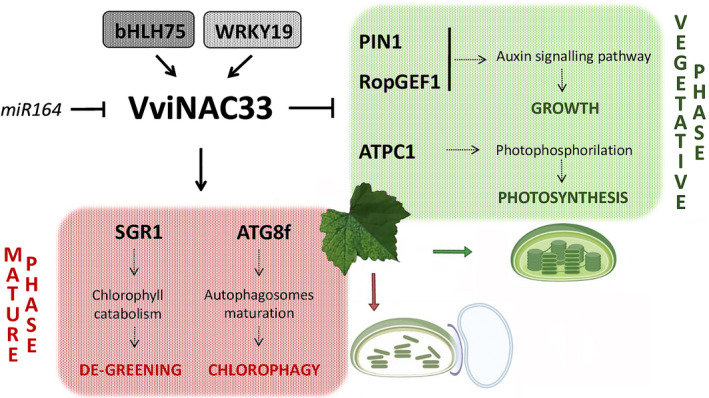
Proposed regulatory network model of the VviNAC33 mechanisms of action. Induced and repressed VviNAC33 targets identified in this work and the biological processes they are involved in, are indicated together with the transcription factors controlling *VviNAC33* expression. The negative regulation of *VviNAC33* by *miR164* has been demonstrated by Sun *et al*. (2012). The figure also shows the chloroplast images corresponding to vegetative and mature phase (lower right corner). The chloroplast images were created with biorender (https://biorender.com/).

Our data defined VviNAC33 as a central hub in a regulatory network that controls de‐greening and growth of grapevine organs during the vegetative‐to‐mature phase transition (Fig. 10). This represents a key step towards a comprehensive understanding of the intricate molecular circuits that combine developmental and environmental cues in grapevine.

## Author contributions

ED, SC, CF and EB performed the research. ED, SC and SZ analysed the data. MG, AG and NV contributed new analytic and computational tools. SZ, GBT and MP designed the research. SZ, ED and GBT wrote the paper.

## Supporting information


**Dataset S1** VviNAC33 co‐expression analysis in the Corvina atlas.

**Dataset S2** DNA affinity purification sequencing (DAP‐seq) unfiltered dataset.

**Dataset S3** DNA affinity purification sequencing (DAP‐seq) dataset after removing peaks with a sample : control ratio < 5 fold from Dataset S2.

**Dataset S4** Dataset of transient overexpressing VviNAC33 transgenic plants and controls.

**Dataset S5** Dataset of stable overexpressing VviNAC33 transgenic plants and controls.

**Fig. S1** *VviNAC33* expression during berry development.
**Fig. S2** Alignment of VviNAC33 predicted amino acidic sequences from *Vitis* *vinifera* cv Pinot Noir and cv Corvina.
**Fig. S3** Phylogenetic analysis of the 74 grapevine NAC genes.
**Fig. S4** Heat map of NAC members expressed in grapevine organs.
**Fig. S5** Bioinformatics analysis of the VviNAC33 protein domains.
**Fig. S6** RSAT Plants NGS ChIP‐Seq peak motif analysis and dyad analysis.
**Fig. S7** *VviNAC33* expression level determined by qPCR in transgenic grapevine leaves (*Vitis* *vinifera* cv Sultana).
**Fig. S8** Transient overexpression of *VviNAC33* accelerates de‐greening in *Nicotiana* *benthamiana* leaves.
**Fig. S9** Transgene expression levels, copy numbers in transgenic grapevine lines overexpressing *VviNAC33* and leaf area measurement.
**Fig. S10** Analysis of photosynthetic parameters in transgenic grapevine lines overexpressing *VviNAC33*.
**Fig. S11** Effect of 1‐naphthaleneacetic acid (NAA) at different concentrations (5 and 20 mg l^−1^) on OX2 and control lines of *in* *vitro* stems consisting of two apical leaves (one fully expanded and the other newly developing) at 14 d after hormone treatment.
**Fig. S12** Transgene expression levels, copy numbers in transgenic grapevine lines expressing *VviNAC33‐EAR* and leaf area measurement.
**Fig. S13** Pigment content and PSII fluorescence in transgenic grapevine lines expressing *VviNAC33‐EAR*.
**Fig. S14** Predicted VviNAC33 binding sites in promoter sequences.
**Fig. S15** *VviNAC33* upstream regulation.
**Table S1** List of the primers used in this study.

**Table S2** VCost.v3 gene annotation of 74 grapevine NAC transcription factors.
**Table S3** Differentially expressed genes identified by transient expression and in transgenic plants overexpressing VviNAC33.
**Table S4** Fold changes in expression (transient expression and stable transgenic leaves) of the 139 putative direct targets of VviNAC33, and expression trends in developing leaves.
**Table S5** Genes involved in auxin signaling and metabolism differentially expressed in leaves overexpressing VviNAC33and/or identified by DAP‐seq analysis.
Please note: Wiley Blackwell are not responsible for the content or functionality of any Supporting Information supplied by the authors. Any queries (other than missing material) should be directed to the *New* *Phytologist* Central Office.

## Data Availability

Microarray data for the transient expression experiments are available at GEO under the series entry GSE155037 (https://www.ncbi.nlm.nih.gov/geo/query/acc.cgi?acc=GSE155037). Microarray data for the transgenic plants are available at GEO under the series entry GSE156105 **(** https://www.ncbi.nlm.nih.gov/geo/query/acc.cgi?acc=GSE156105). DAP‐seq data are available at GEO under the series entry GSE155445 **(** https://www.ncbi.nlm.nih.gov/geo/query/acc.cgi?acc=GSE155445).
