# Supplementary material for: VviNAC33 promotes organ de‐greening and represses vegetative growth during the vegetative‐to‐mature phase transition in grapevine
Source: New Phytol. 2021 Mar 16;231(2):726–46. doi: 10.1111/nph.17263 (PMC8251598; doi:10.1111/nph.17263)
Supplement: Supplementary file 6 — Fig. S1 VviNAC33 expression during berry development. Fig. S2 Alignment of VviNAC33 predicted amino acidic sequences from Vitis vinifera cv Pinot Noir and cv Corvina. Fig. S3 Phylogenetic analysis of the 74 grapevine NAC genes. Fig. S4 Heat map of NAC members expressed in grapevine organs. Fig. S5 Bioinformatics analysis of the VviNAC33 protein domains. Fig. S6 RSAT Plants NGS ChIP‐Seq peak motif analysis and dyad analysis. Fig. S7 VviNAC33 expression level determined by qPCR in transgenic grapevine leaves (Vitis vinifera cv Sultana). Fig. S8 Transient overexpression of VviNAC33 accelerates de‐greening in Nicotiana benthamiana leaves. Fig. S9 Transgene expression levels, copy numbers in transgenic grapevine lines overexpressing VviNAC33 and leaf area measurement. Fig. S10 Analysis of photosynthetic parameters in transgenic grapevine lines overexpressing VviNAC33. Fig. S11 Effect of 1‐naphthaleneacetic acid (NAA) at different concentrations (5 and 20 mg l−1) on OX2 and control lines of in vitro stems consisting of two apical leaves (one fully expanded and the other newly developing) at 14 d after hormone treatment. Fig. S12 Transgene expression levels, copy numbers in transgenic grapevine lines expressing VviNAC33‐EAR and leaf area measurement. Fig. S13 Pigment content and PSII fluorescence in transgenic grapevine lines expressing VviNAC33‐EAR. Fig. S14 Predicted VviNAC33 binding sites in promoter sequences. Fig. S15 VviNAC33 upstream regulation. Table S1 List of the primers used in this study. [file NPH-231-726-s007.pdf]

New Phytologist Supporting Information

Article title: VviNAC33 promotes organ de-greening and represses vegetative growth during the vegetative-to-mature phase transition in grapevine

Authors: Erica D'Incà, Stefano Cazzaniga, Chiara Foresti, Nicola Vitulo, Edoardo Bertini, Mary Galli, Andrea Gallavotti, Mario Pezzotti, Giovanni Battista Tornielli and Sara Zenoni

Article acceptance date: 1 February 2021

Fig. S1 *VviNAC33* expression during berry development. (a) *VviNAC33* expression profile in 10 different grapevine varieties at four developmental stages (Massonnet M, Fasoli M, Tornielli GB, Altieri M, Sandri M, Zuccolotto P, Paci P, Gardiman M, Zenoni S, Pezzotti M. 2017. Ripening transcriptomic program in red and white grapevine varieties correlates with berry skin anthocyanin accumulation. *Plant Physiology* 174(4):2376-2396. doi: 10.1104/pp.17.00311. PMID: 28652263; PMCID: PMC5543946.) and (b) in cv. Cabernet Sauvignon berries during development, sampled every 10 days from fruit set to ripening (Fasoli M, Richter CL, Zenoni S, Bertini E, Vitulo N, Dal Santo S, Dokoozlian N, Pezzotti M, Tornielli GB. 2018. Timing and order of the molecular events marking the onset of berry ripening in grapevine. *Plant Physiology*. 178(3):1187-1206. doi: 10.1104/pp.18.00559. PMID: 30224433; PMCID: PMC6236592). (c) *VviNAC33* genotype x environment (GxE) cluster of gene expression (Dal Santo S, Zenoni S, Sandri M, De Lorenzis G, Magris G, De Paoli E, Di Gaspero G, Del Fabbro C, Morgante M, Brancadoro L, Grossi D, Fasoli M, Zuccolotto P, Tornielli GB, Pezzotti M. 2018. Grapevine field experiments reveal the contribution of genotype, the influence of environment and the effect of their interaction (G×E) on the berry transcriptome. *The Plant journal: for cell and molecular biology* 93(6), 1143–1159. https://doi.org/10.1111/tpj.13834). (d) Box plot of variable importance measure (VIM) of the cluster represented in (c) used to characterize the relationship between the cluster and the four experimental conditions. (Dal Santo S, Zenoni S, Sandri M, De Lorenzis G, Magris G, De Paoli E, Di Gaspero G, Del Fabbro C, Morgante M, Brancadoro L, Grossi D, Fasoli M, Zuccolotto P, Tornielli GB, Pezzotti M. 2018. Grapevine field experiments reveal the contribution of genotype, the influence of environment and the effect of their interaction (G×E) on the berry transcriptome. *The Plant journal: for cell and molecular biology* **93(6)**, 1143–1159. https://doi.org/10.1111/tpj.13834).

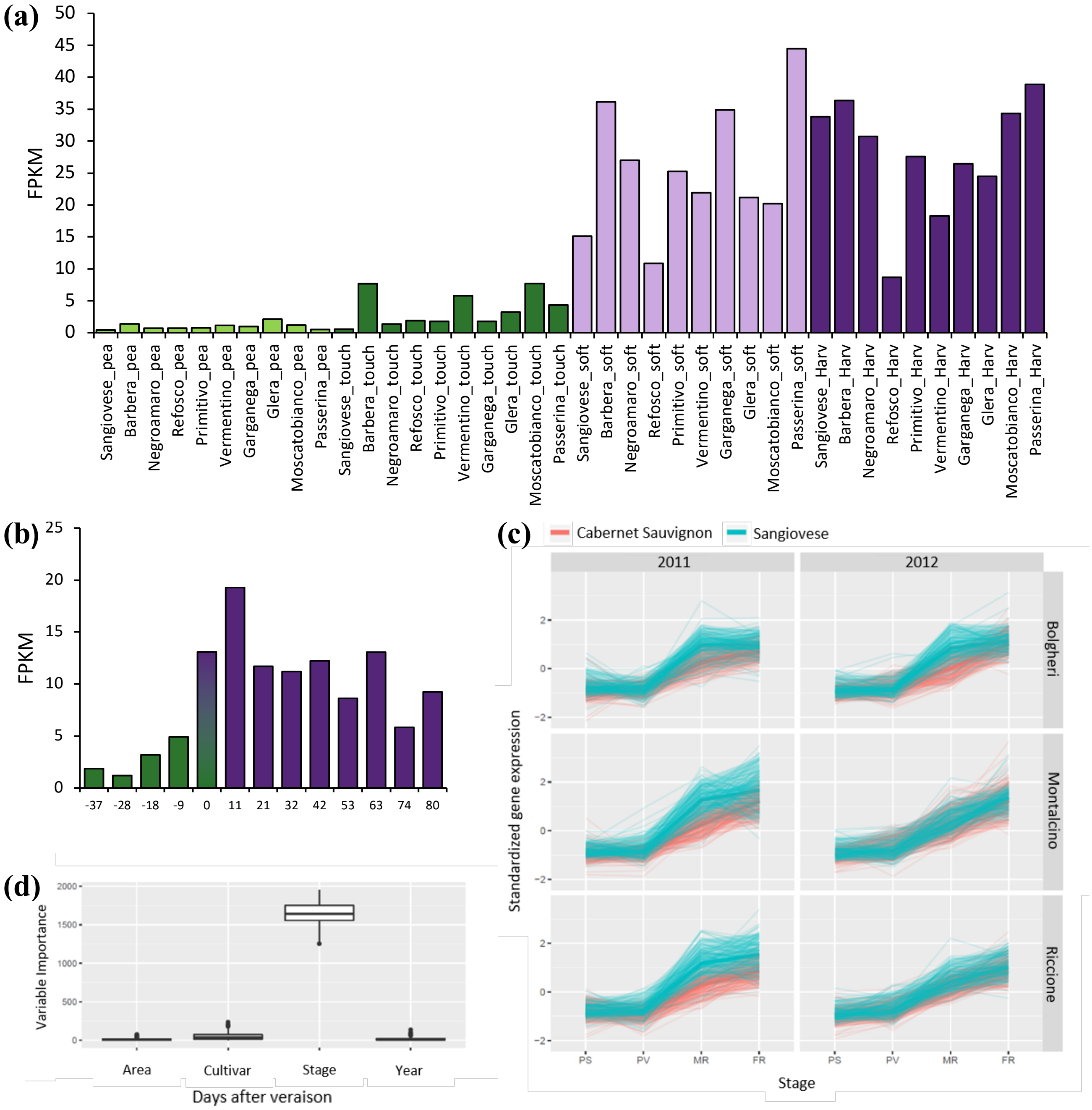
(a)
FPKM
50
45
40
35
30
25
20
15
10
5
0
Sangiovese_pea
Barbera_pea
Negroamaro_pea
Refosco_pea
Primitivo_pea
Vermentino_pea
Garganega_pea
Glera_pea
Moscatobianco_pea
Passerina_pea
Sangiovese_touch
Barbera_touch
Negroamaro_touch
Refosco_touch
Primitivo_touch
Vermentino_touch
Garganega_touch
Glera_touch
Moscatobianco_touch
Passerina_touch
Sangiovese_soft
Barbera_soft
Negroamaro_soft
Refosco_soft
Primitivo_soft
Vermentino_soft
Garganega_soft
Glera_soft
Moscatobianco_soft
Passerina_soft
Sangiovese_Harv
Barbera_Harv
Negroamaro_Harv
Refosco_Harv
Primitivo_Harv
Vermentino_Harv
Garganega_Harv
Glera_Harv
Moscatobianco_Harv
Passerina_Harv
(b)
FPKM
25
20
15
10
5
0
-37
-28
-18
-9
0
11
21
32
42
53
63
74
80
Days after veraison
(c)
Cabernet Sauvignon
Sangiovese
2011
2012
Bolgheri
Montalcino
Riccione
Standardized gene expression
2
0
-2
PS
PV
MR
FR
Stage
(d)
Variable Importance
2000
1500
1000
500
0
Area
Cultivar
Stage
Year

Fig. S2 Alignment of VviNAC33 predicted amino acidic sequences from cv. Pinot Noir and cv. Corvina. The amino acid substitution is labelled in red. The black line above the alignment shows the nuclear localization signal (NLS) predicted using PSORTII.

```
Pinot Noir    MVESRLPPGFRFHPKDEELICDYLMKKVTSSESSLFIEVDLNKCEPWDIPEMACVGSKDW    60
Corvina       MVESRLPPGFRFHPKDEELICDYLMKKVTSSESSLFIEVDLNKCEPWDIPEMACVGSKDW    60
              ************************************************************

                                                                    NLS
Pinot Noir    YFYNQRDRKYATGLRTNRATLSGYWKATGKDRPILSKGTLVGMRKTLVFYQGRAPKGKKT    120
Corvina       YFYNQRDRKYATGLRTNRATLSGYWKATGKDRPILSKGTLVGMRKTLVFYQGRAPKGKKT    120
              ************************************************************

Pinot Noir    DWVMHEFRLQGPLTPPAIPSLKEDWVLCRVFNKSRSEAAGKAITSNMGNGYYDNMNMGSS    180
Corvina       DWVMHEFRLQGPLTPPAIPSLKEDWVLCRVFNKSRSEAAGKAITSNMGNGYYDNMNMGSS    180
              ************************************************************

Pinot Noir    TLPPLVDSYINFDQTEIKLNEYEQVPCFSDMCSPNPSNLVFPHITHLEPHLLTKTIAPIF    240
Corvina       TLPPLVDSYINFDQTEIKLNEYEQVPCFSDMCSPNPSNLVFPHITHMEPHLLTKTIAPIF    240
              ********************************************** *************

Pinot Noir    GGMPDLGTFSCDKMVIKTVLNQLSNVEESPSFGEGSSSESYLSEVALPPIWNHYC*        295
Corvina       GGMPDLGTFSCDKMVIKTVLNQLSNVEESPSFGEGSSSESYLSEVALPPIWNHYC*        295
              ********************************************************
```

Fig. S3 Phylogenetic analysis of the 74 grapevine NAC genes. The previous clade designation (Wang N, Zheng Y, Xin H, Fang L, Li S. 2013. Comprehensive analysis of NAC domain transcription factor gene family in Vitis vinifera. *Plant Cell Reports* 32(1):61-75. doi: 10.1007/s00299-012-1340-y. PMID: 22983198) is shown as a circle of different colours.

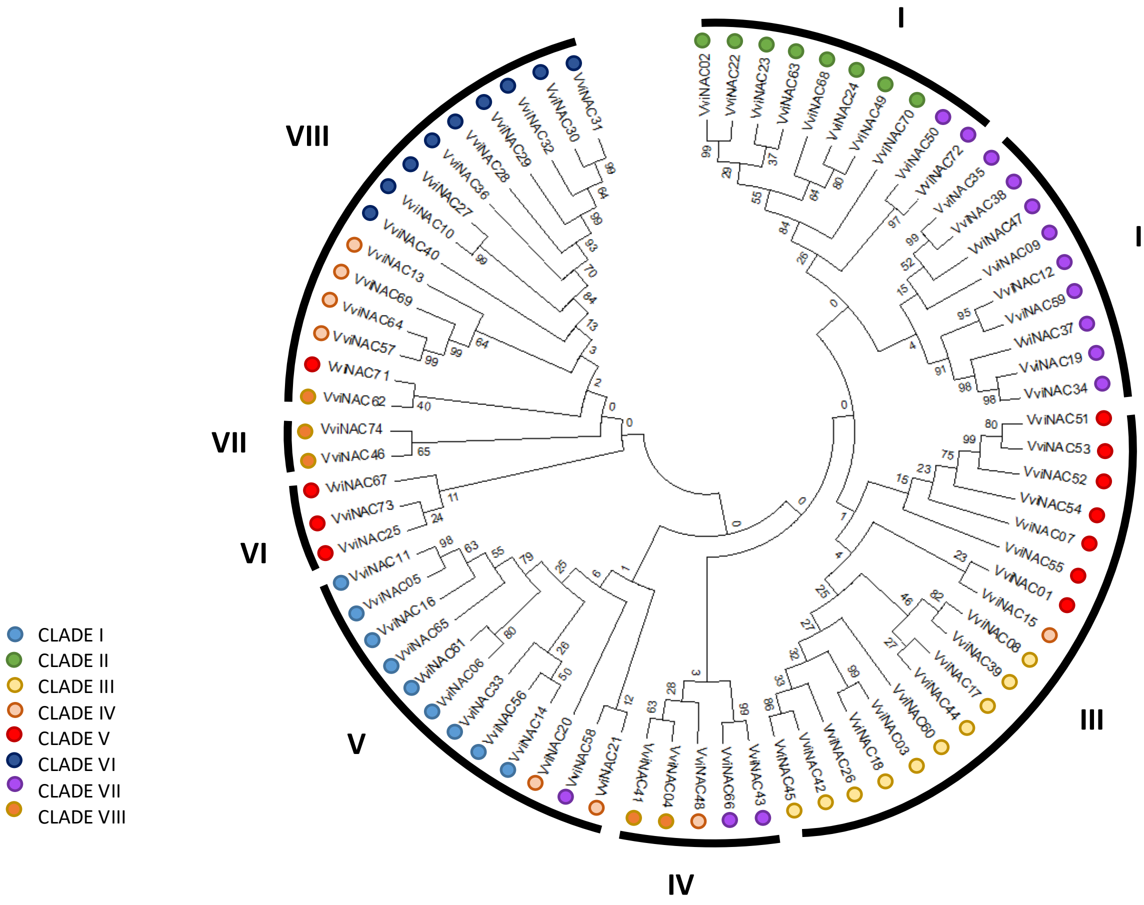

Fig. S4 Heat map of NAC members in grapevine organs. The data were sourced from the atlas transcriptomic dataset (Fasoli M, Dal Santo S, Zenoni S, Tornielli GB, Farina L, Zamboni A, Porceddu A, Venturini L, Bicego M, Murino V, Ferrarini A, Delledonne M, Pezzotti M. 2012. The grapevine expression atlas reveals a deep transcriptome shift driving the entire plant into a maturation program. *Plant Cell* 24(9):3489-505. doi: 10.1105/tpc.112.100230. PMID: 22948079; PMCID: PMC3480284).

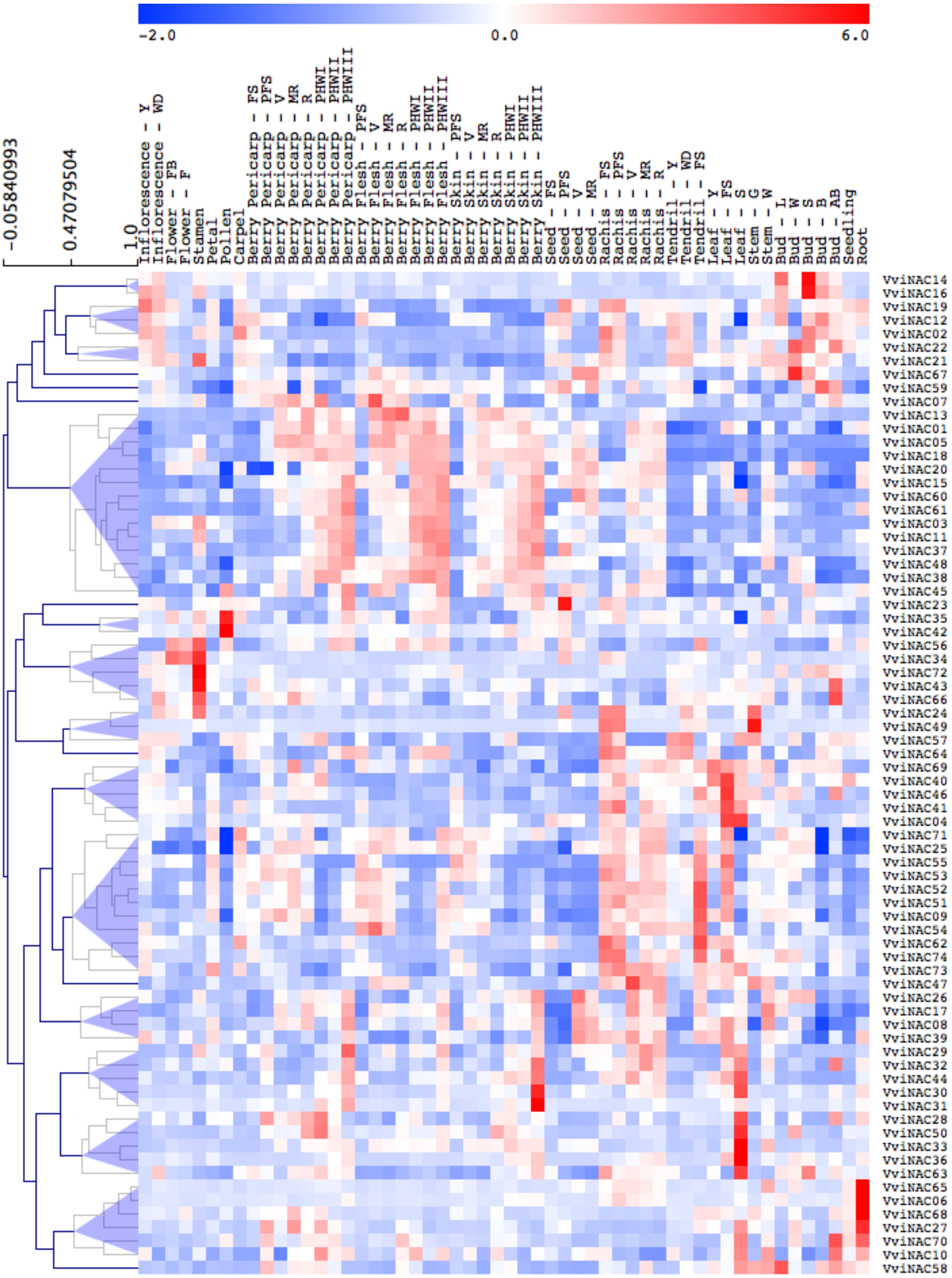

-2.0
0.0
6.0
-0.05840993
0.47079504
1.0
Inflorescence - Y
Inflorescence - WD
Flower - FB
Flower - F
Stamen
Petal
Pollen
Carpel
Berry Pericarp - FS
Berry Pericarp - PFS
Berry Pericarp - V
Berry Pericarp - MR
Berry Pericarp - R
Berry Pericarp - PHWI
Berry Pericarp - PHWII
Berry Pericarp - PHWIII
Berry Flesh - PFS
Berry Flesh - V
Berry Flesh - MR
Berry Flesh - R
Berry Flesh - PHWI
Berry Flesh - PHWII
Berry Flesh - PHWIII
Berry Skin - PFS
Berry Skin - V
Berry Skin - MR
Berry Skin - R
Berry Skin - PHWI
Berry Skin - PHWII
Berry Skin - PHWIII
Seed - FS
Seed - PFS
Seed - V
Seed - MR
Rachis - FS
Rachis - PFS
Rachis - V
Rachis - MR
Rachis - R
Tendril - Y
Tendril - WD
Tendril - FS
Leaf - Y
Leaf - FS
Leaf - S
Stem - G
Stem - W
Bud - L
Bud - W
Bud - S
Bud - B
Bud - AB
Seedling
Root
VviNAC14
VviNAC16
VviNAC19
VviNAC12
VviNAC02
VviNAC22
VviNAC21
VviNAC67
VviNAC59
VviNAC07
VviNAC13
VviNAC01
VviNAC05
VviNAC18
VviNAC20
VviNAC15
VviNAC60
VviNAC61
VviNAC03
VviNAC11
VviNAC37
VviNAC48
VviNAC38
VviNAC45
VviNAC23
VviNAC35
VviNAC42
VviNAC56
VviNAC34
VviNAC72
VviNAC43
VviNAC66
VviNAC24
VviNAC49
VviNAC57
VviNAC64
VviNAC69
VviNAC40
VviNAC46
VviNAC41
VviNAC04
VviNAC71
VviNAC25
VviNAC55
VviNAC53
VviNAC52
VviNAC51
VviNAC09
VviNAC54
VviNAC62
VviNAC74
VviNAC73
VviNAC47
VviNAC26
VviNAC17
VviNAC08
VviNAC39
VviNAC29
VviNAC32
VviNAC44
VviNAC30
VviNAC31
VviNAC28
VviNAC50
VviNAC33
VviNAC36
VviNAC63
VviNAC65
VviNAC06
VviNAC68
VviNAC27
VviNAC70
VviNAC10
VviNAC58

Fig. S5 Bioinformatics analysis of the VviNAC33 protein domains. The sequence logos of the seven motifs detected in proteins belonging to the VviNAC33 clade in the phylogenetic tree were identified using MEME software. The bit score indicates the information content for each position in the sequence.

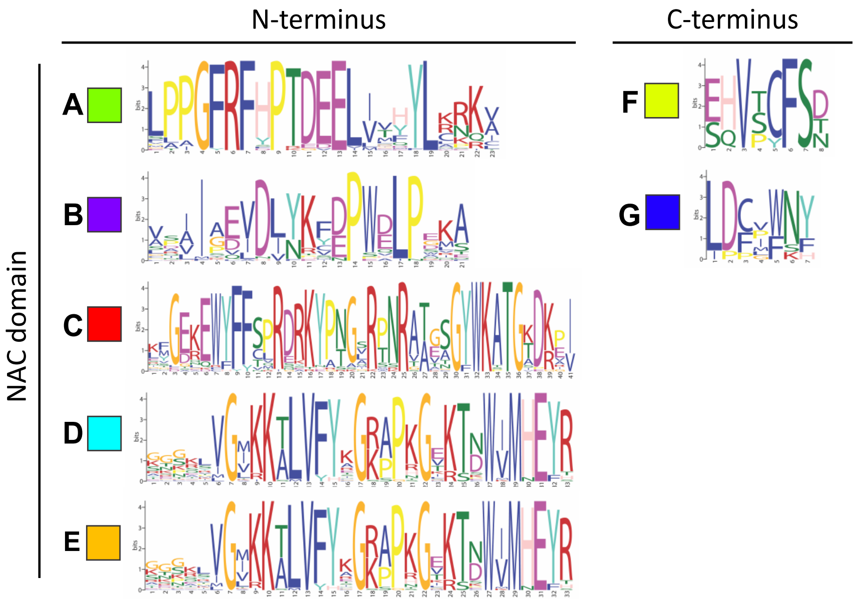

Fig. S6 RSAT Plants NGS ChIP-Seq peak motifs analysis and dyad analysis. (a) Three top-ranking binding motifs identified in the DAP-Seq filtered dataset for the detection of overrepresented oligonucleotides. (b) Top-ranking binding motifs identified in the DAP-Seq filtered dataset focusing on the promoter and 5′ UTR, based on the detection of overrepresented oligonucleotides (oligo analysis) and overrepresented spaced pairs of oligonucleotides (dyad analysis). The overall height of each letter stack indicates the sequence conservation at that position, and the height of symbols within the stack reflects the relative frequency of the corresponding nucleic acid at that position. The *k-mer sig* is the $-\log_{10}$ of the e value (higher values are associated with more significant patterns). The e value represents the number of patterns with the same degree of overrepresentation expected by chance alone, and is obtained by multiplying the p value by the number of distinct patterns.

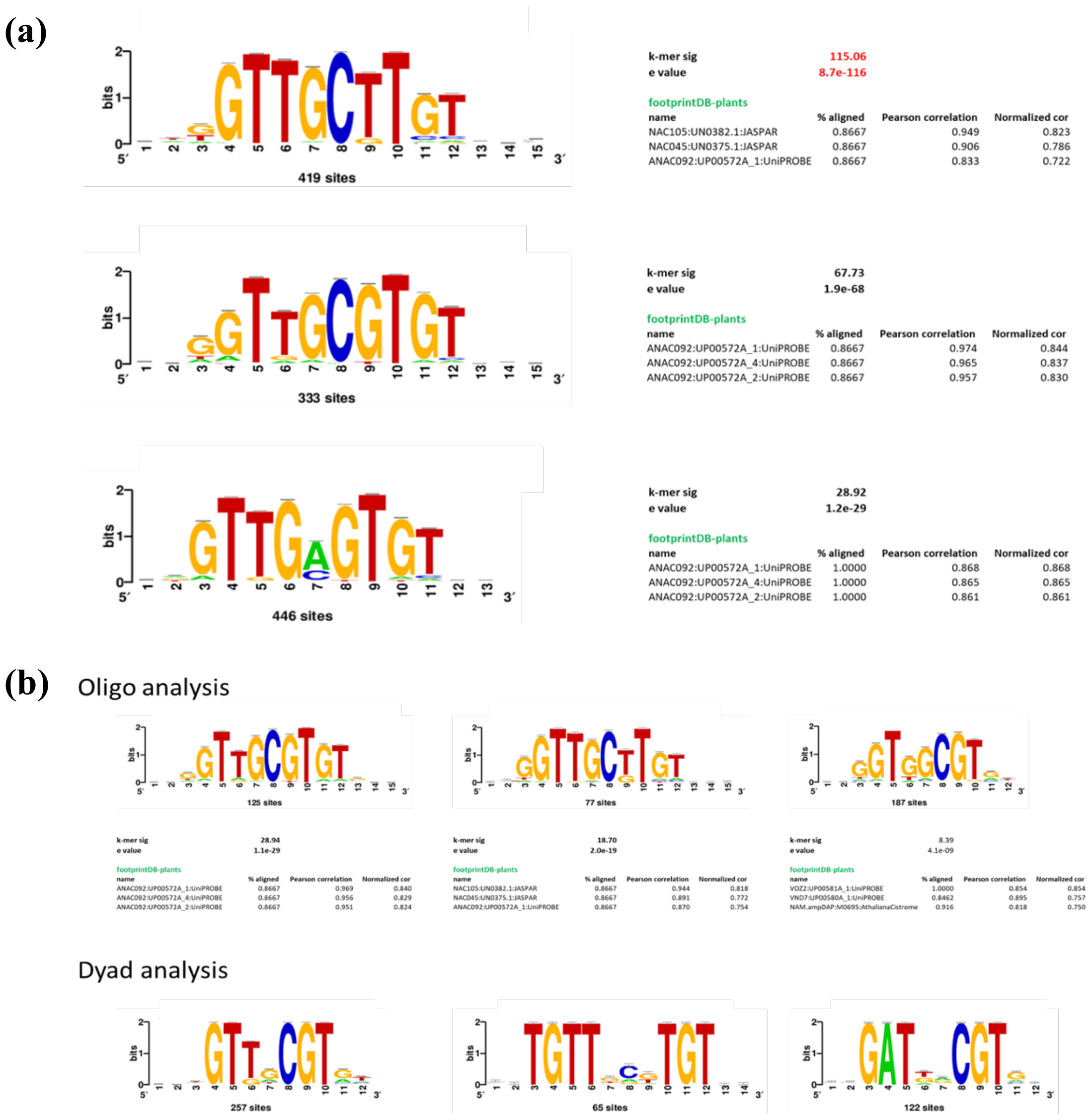

Fig. S7 *VviNAC33* expression level determined by qPCR in transgenic grapevine leaves (cv. Sultana). Each value corresponds to the mean ± standard deviation (SD) of three technical replicates relative to the *VviUBIQUITIN1* (VIT_16s0098g01190) control. C1–C7 = control lines; #1–#8 = OXNAC33 transgenic lines. Asterisks (*) indicate the selected lines for further analysis.

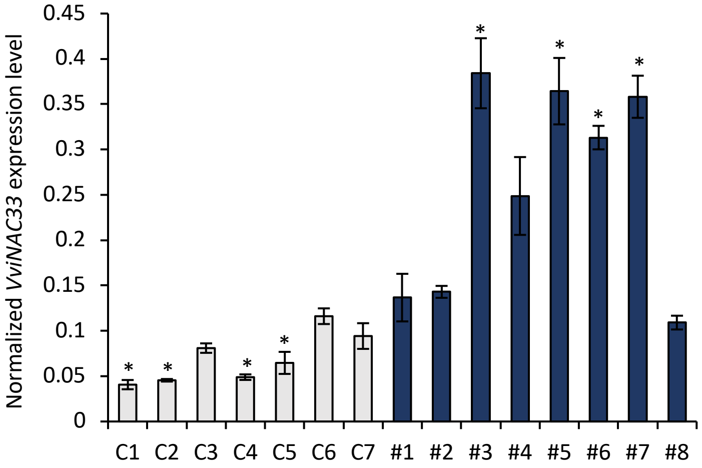

Fig. S8 Transient overexpression of *VviNAC33* accelerates de-greening in *Nicotiana benthamiana* leaves. (a) Phenotype of *N. benthamiana* leaves infiltrated with 35S:*VviNAC33* (OX) and the vector control (C) 3 days post-infiltration. (b) Chlorophyll (Chl) content and (c) chlorophyll/carotenoid (Chl/Car) ratio. (d) PSII maximum quantum efficiency (F_v/F_m). All data are expressed as mean ± standard deviation (SD, n = 4). Asterisks (*) indicate significant differences (*t*-test; $p < 0.05$) between OX and C.

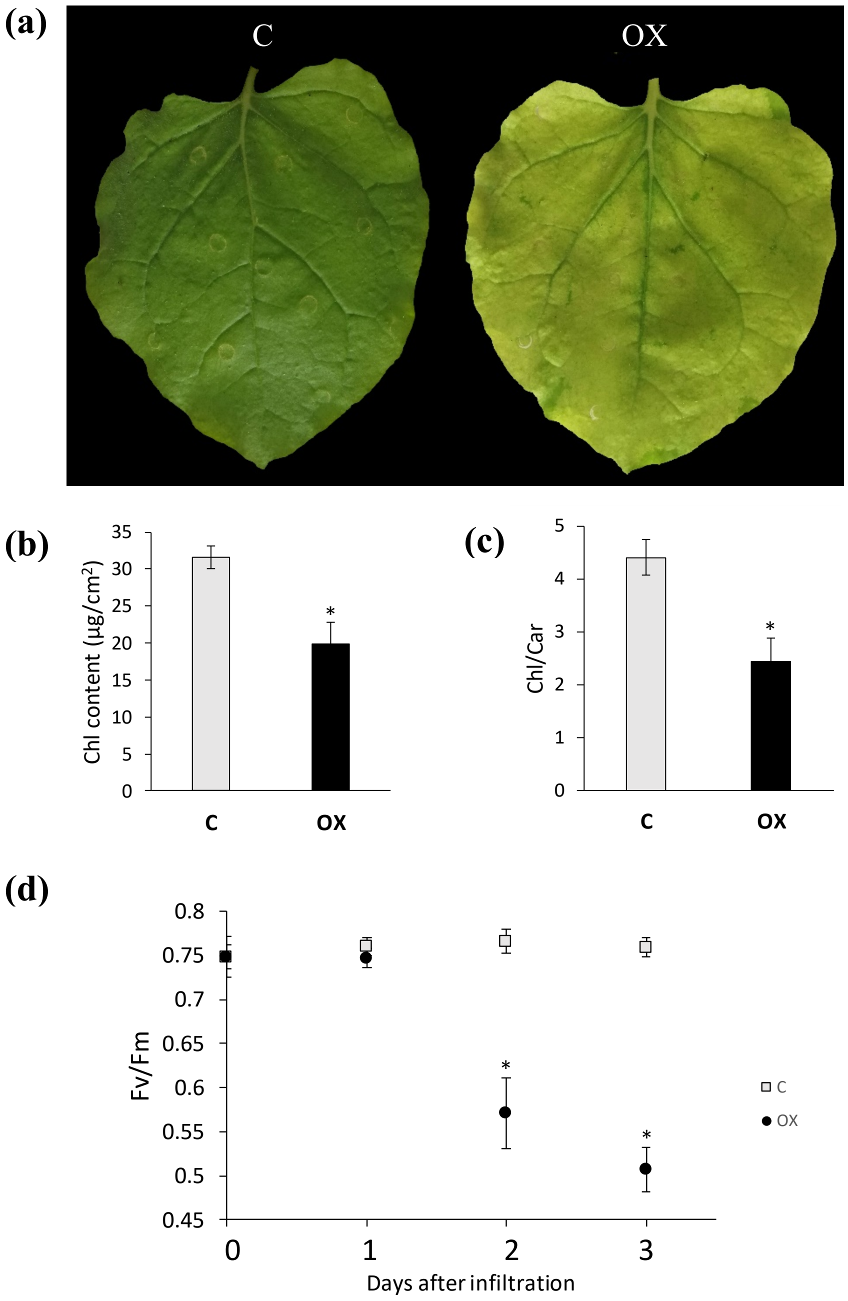

Fig. S9 Transgene expression levels, copy numbers in transgenic grapevine lines overexpressing *VviNAC33* and leaf area measurement. (a) *VviNAC33* expression level in transgenic grapevine leaves at the onset of phenotypic changes, determined by qPCR. C1–C3 = control lines; #1–#10 = OXNAC33 transgenic lines. All data are expressed as mean ± standard deviation (SD, n = 3). We selected #4 (OX1), #5 (OX2) and #8 (OX3) for further analysis (Figs. 5 and 6). (b) Phenotypes of transgenic leaves compared to a vector control. The severity of the phenotype correlated with the *VviNAC33* overexpression level. (c) Southern blot analysis (Walker AR, Lee E, Bogs J, McDavid DA, Thomas MR, Robinson SP. 2007. White grapes arose through the mutation of two similar and adjacent regulatory genes. *Plant Journal* 49(5):772-85. doi: 10.1111/j.1365-313X.2006.02997.x. PMID: 17316172) to determine the transgene copy number. L = 1 kb ladder; C = control line; OX1–3 = lines overexpressing *VviNAC33*; WT = wild-type *V. vinifera* cv. Shiraz plant (negative control to exclude non-specific signals); + refers to the GFP probe (positive control). (d) Relative leaf area measurement of OXNAC33 transgenic lines compared to the control (n = 12 ± SD). SD, standard deviation. Asterisks (*) indicate significant differences (*t*-test; p < 0.01) in the OXNAC33 lines compared to the vector control.

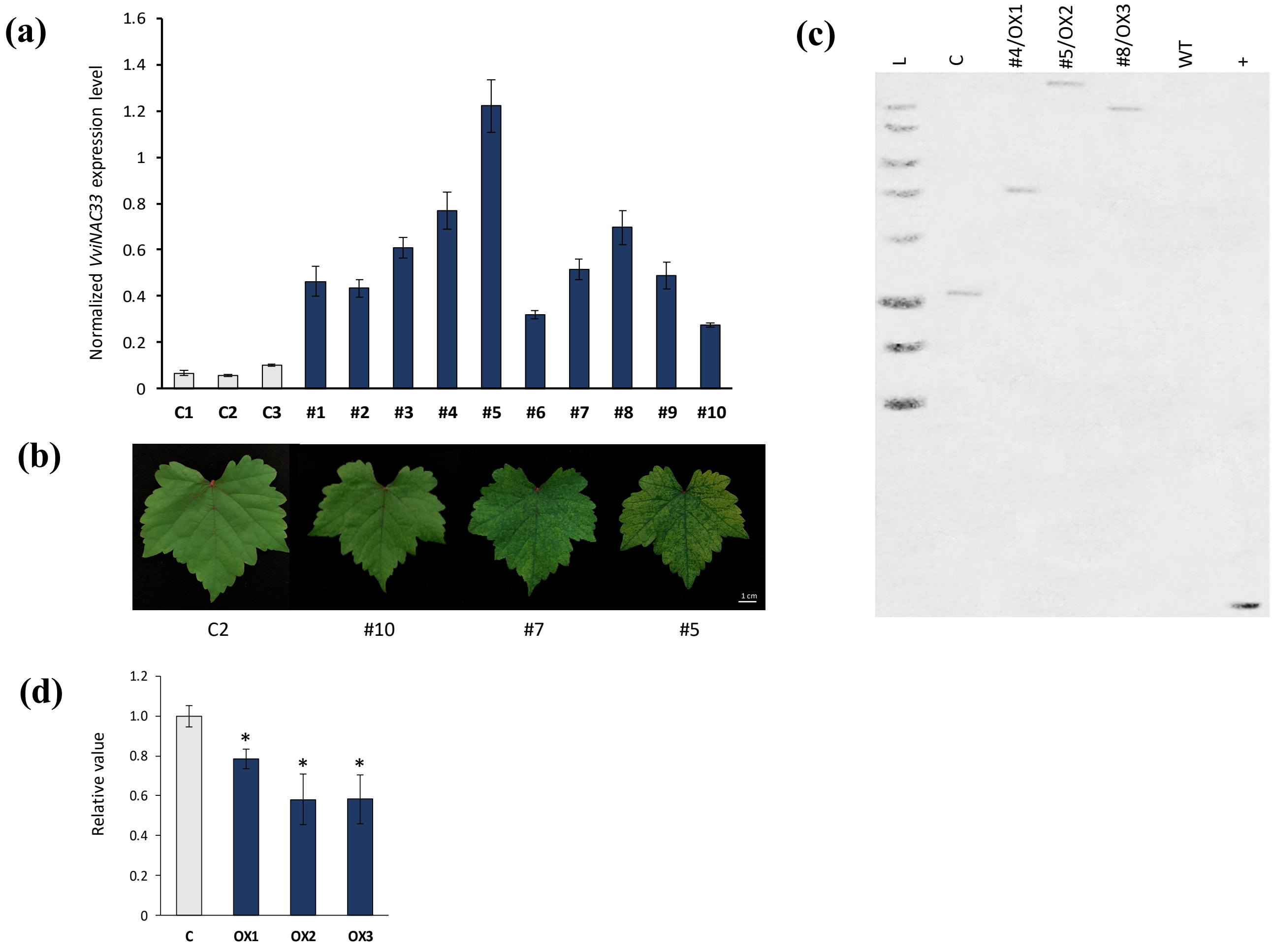

Fig. S10 Analysis of photosynthetic parameters in transgenic grapevine lines overexpressing *VviNAC33*. (a) Non-photochemical quenching (NPQ) of actinic light intensity for the three OXNAC33 lines and the vector control. Data are expressed as mean ± standard deviation (SD, n = 4). Asterisks (*) indicate significant differences (*t*-test; $p < 0.05$) between the OXNAC33 lines and control. (b) Kinetics of the OJIP fluorescence induction curve in the three OXNAC33 lines and control. The values represent the average of at least six independent leaves.

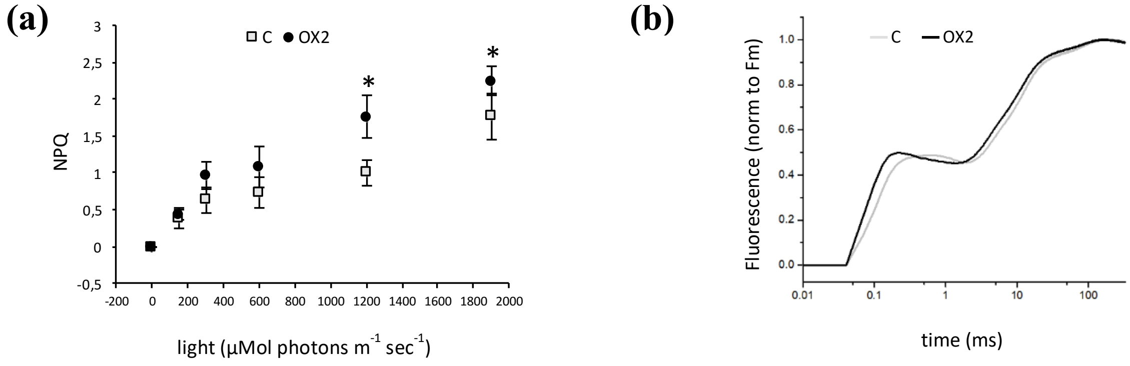

Fig. S11 Effect of 1-Naphthaleneacetic acid (NAA) at different concentrations (5 and 20 mg/L) on OX2 and control lines of *in vitro* stems consisting of two apical leaves (one fully expanded and the other newly developing) at 14 days after hormone treatment.

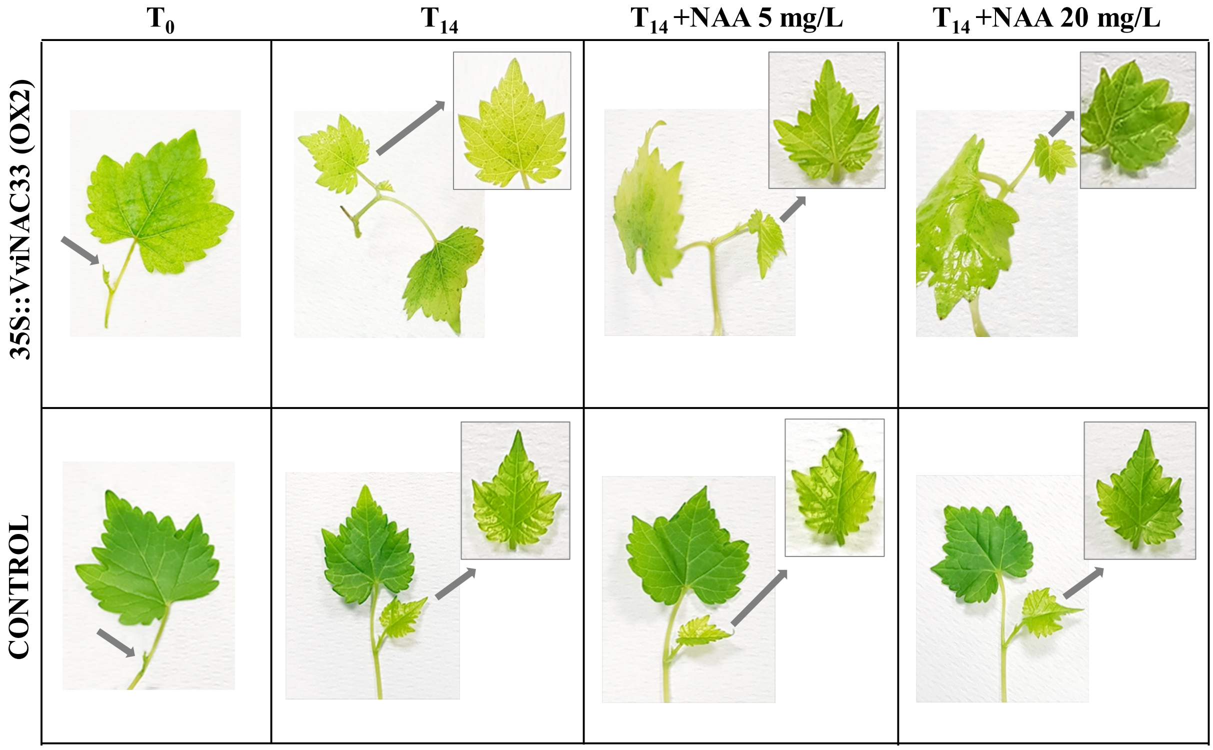

Fig. S12 Transgene expression levels, copy numbers in transgenic grapevine lines expressing *VviNAC33-EAR* and leaf area measurement. (a) RT-PCR analysis of *VviNAC33* transcripts in fully-expanded leaves. L = 1 kb ladder; #1–#4 = EARNAC33 lines; C = control line; + represents the positive control plasmid; – represents the modified pK7WG2 negative control plasmid. We selected #1 (EAR1), #2 (EAR2) and #4 (EAR3) for further analysis (Fig. 8a). (b) Southern blot analysis (Walker AR, Lee E, Bogs J, McDavid DA, Thomas MR, Robinson SP. 2007. White grapes arose through the mutation of two similar and adjacent regulatory genes. *Plant Journal* 49(5):772-85. doi: 10.1111/j.1365-313X.2006.02997.x. PMID: 17316172) to determine the transgene copy number. L = 1 kb ladder; EAR1–3 = EARNAC33 lines; C = control line; WT = wild-type *V. vinifera* cv. Garganega plant (negative control to exclude non-specific signals); + refers to the GFP probe (positive control). (c) Relative leaf area measurement of VviNAC33-EAR transgenic lines compared to the control (n = 12 ± SD). SD, standard deviation. Asterisks (*) indicate significant differences (*t*-test; p < 0.01) in the VviNAC33-EAR lines compared to the vector control.

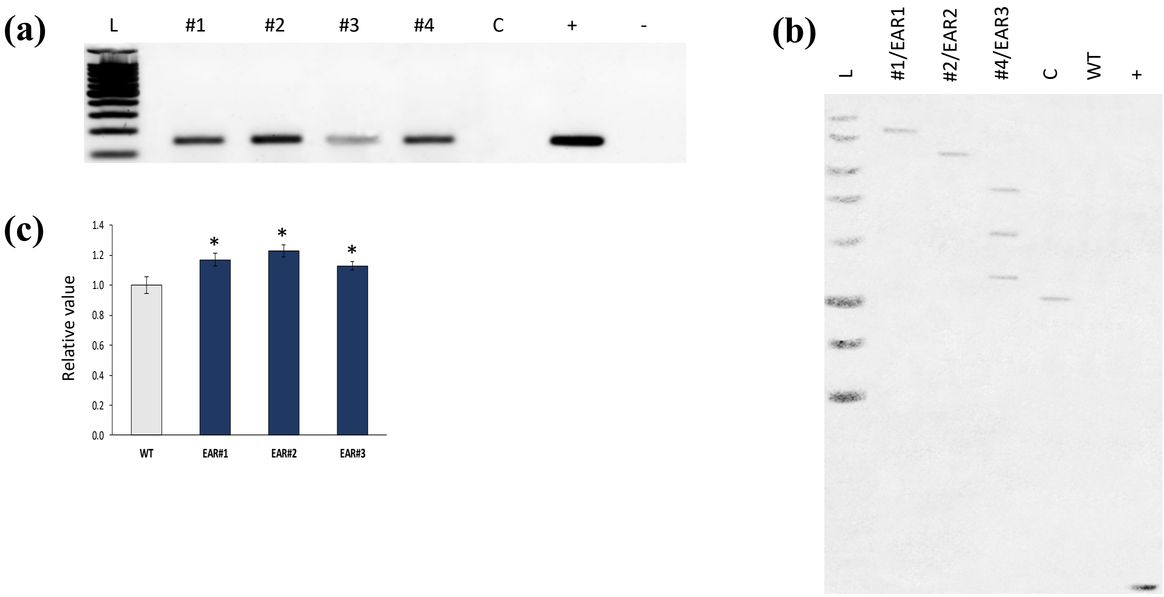

Fig. S13 Pigment content and PSII fluorescence in transgenic grapevine lines expressing VviNAC33-EAR. (a) Chlorophyll a and b (Chl a/b) and Chl carotenoid ratio (Chl/Car). (b) PSII maximum quantum efficiency (Fv/Fm). (c) F0 and Fm normalized to Chl. All data are expressed as mean ± SD. (n = 4). SD, standard deviation.

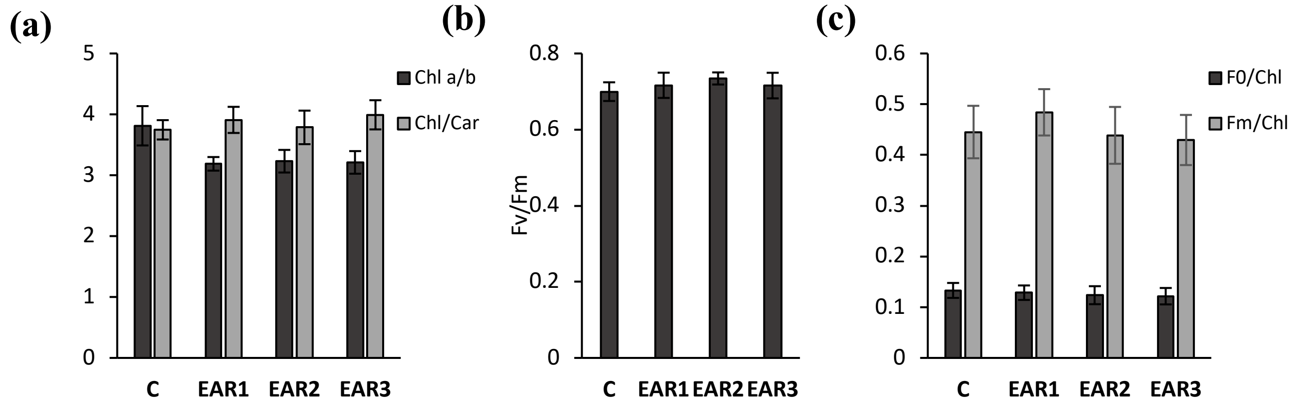

Fig. S14 Predicted VviNAC33-binding sites in promoter sequences. Promoter sequences are shown for the genes *SGR1, ATG8F, PIN1, RopGEF1* and *ATPC1*. The sequences were isolated from *V. vinifera* cv. Corvina and cloned in vector pPGWL7.0 for the Dual Luciferase Reporter Assay in *Nicotiana benthamiana* leaves. The three top-ranking binding motifs found by RSAT oligo analysis on the DAP-Seq dataset are shown in red, light blue and black bold within the sequences (as reported in the right corner). The common feature among the three binding motifs is underlined in black. The started codon ATG is highlighted in yellow.

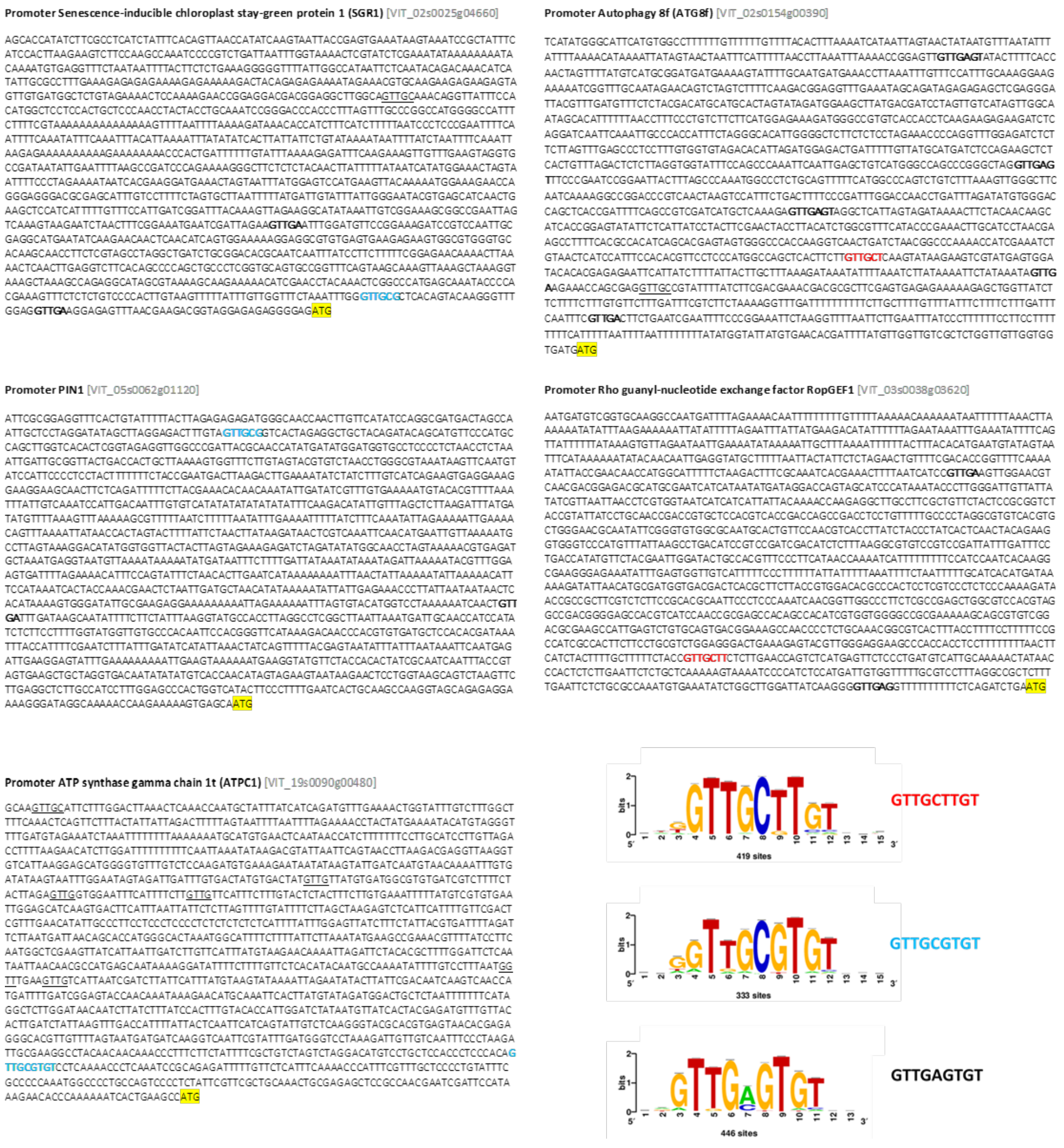

Fig. S15 VviNAC33 upstream regulation. The activity of VviNAC33 promoter was tested in the presence and absence of the 35S:VvibHLH75 and 35S:VviWRKY19 effector vectors. LUC values are reported relative to the REN value and normalized against the control (empty effector vector). LUC values represent the mean of four biological replicates ± standard deviation (SD). Asterisks (*) indicate significant differences in promoter activation compared with the control (*t*-test; $p < 0.01$).

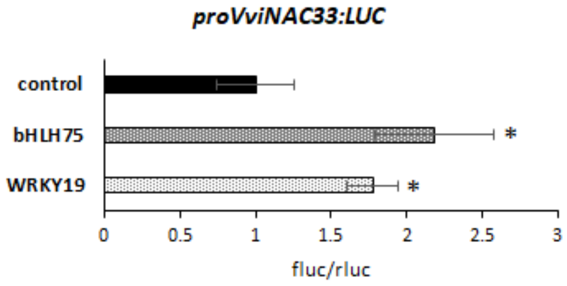

Table S1 List of the primers used in this study.

GENE		SEQUENCE 5'-3'
ATG8f	qPCR	CATCTTTGCCTGCATCGTGA<br>GAAGCAAGAGTTTCTAACATCG
	Promoter Isolation	CACCTCATATGGGCATTCATGTGGC<br>CATCACCACCAACAACCAGA
ATPC1	qPCR	GCAGGCCAAAATCACAGGTG<br>TCTCATCTAAGAAGCCCAAGC
	Promoter Isolation	CACCGCAAGTTGCATTCTTTGGACT<br>CGAATAGAGGGGACTGGCA
PIN1	qPCR	AGCCATCCTGGTGATTGATCA<br>TGTGATGTCTGTGGTGTGCA
	Promoter Isolation	CACCATTCGCGGAGGTTTCACTGT<br>TGCTCACTTTTTCTTGGTTTTTG
RopGEF1	qPCR	GCTAGATGAAGAGAGTTATGCT<br>TTACATCCAGCTAGGGCACT
	Promoter Isolation	CACCCAATGATGTCGGTGCAAGGC<br>TCAGATCTGAGAAAAAAAAAACC
SGR1	qPCR	CCTGACCAGATGATCTTTACT<br>CAGTAGCAATGCAAGTGCTTG
	Promoter Isolation	CACCGGGCTTAGGCAACAGACTCTT<br>CTCCCCTCTCTCCTACCGTC
Sucrase	qPCR	TTCTGTCGCCGCTGTGTATT<br>ATCATTCAGCGACCCTAAGG
VviNAC33	Gene Isolation_Overexpressing Plants	CACCATGGTTGAGTCAAGGTTGCCA<br>CTAACAATAATGGTTCCAAATGG
	Gene Isolation_Repressing Plants	CACCATGGTTGAGTCAAGGTTGCCA<br>TTAAGCGAAACCCAAACGGAGTTCTAGATCCAGATCGAGACAATAATGGTTCCAAATGG
	Gene Isolation_Subcellular Localizatic	CACCATGGTTGAGTCAAGGTTGCCA<br>ACAATAATGGTTCCAAATGGGG
	qPCR	TGCCCTGCTTCTCCGATATG<br>CTGGCATTCCTCCAAATATGG
	Promoter Isolation	CCCAAGCTTGGGGTTGGATGGTAAGCATGAAA<br>GGACTAGTCCCTCAATAATGCTCATTTTGA
VviUBIQUITIN	qPCR	TCTGAGGCTTCGTGGTGGTA<br>AGGCGTGCATAACATTTGCG

Table S2 VCost.v3 gene annotation of 74 grapevine NAC transcription factors.

Table S3 Differentially expressed genes identified by transient expression and in transgenic plants overexpressing VviNAC33. The highest fold change (FC) values are highlighted in red, the lowest in blue.

Table S4 Fold changes in expression (transient expression and stable transgenic leaves) of the 139 putative direct targets of VviNAC33, and expression trends in developing leaves. The highest fold change (FC) values are highlighted in red, the lowest in blue.

Table S5 Genes involved in auxin signaling and metabolism differentially expressed in leaves overexpressing VviNAC33and/or identified by DAP-seq analysis. Positive fold change (FC) values are highlighted in red, negative in blues.

Dataset S1 VviNAC33 co-expression analysis in the Corvina atlas. NA: not applicable.

Dataset S2 DNA affinity purification sequencing (DAP-seq) unfiltered dataset.

Dataset S3 DNA affinity purification sequencing (DAP-seq) dataset after removing peaks with a sample/control ratio < 5 fold from Dataset S2.

Dataset S4 Dataset of transient overexpressing VviNAC33 transgenic plants and controls.

Dataset S5 Dataset of stable overexpressing VviNAC33 transgenic plants and controls.
